# Ripples in macaque V1 and V4 are modulated by top-down visual attention

**DOI:** 10.1073/pnas.2210698120

**Published:** 2023-01-25

**Authors:** Jafar Doostmohammadi, Marc Alwin Gieselmann, Jochem van Kempen, Reza Lashgari, Ali Yoonessi, Alexander Thiele

**Affiliations:** ^a^Department of Neuroscience and Addiction Studies, School of Advanced Technologies in Medicine, Tehran University of Medical Sciences, Tehran 13, Iran; ^b^Biosciences Institute, Newcastle University, Newcastle upon Tyne NE1 7RU, United Kingdom; ^c^School of Cognitive Sciences, Institute for Research in Fundamental Sciences, IPM, Tehran 13, Iran; ^d^Institute of Medical Science and Technology, Shahid Beheshti University, Tehran 13, Iran

**Keywords:** attention, sharp wave ripple, visual cortex

## Abstract

Sharp-wave ripples (SWRs) are highly synchronous neuronal activity events. They have been predominantly observed in the hippocampus during offline states such as pause in exploration, slow-wave sleep, and quiescent wakefulness. SWRs have been linked to memory consolidation, spatial navigation, and spatial decision-making. Recently, SWRs have been reported during visual search, a form of remote spatial exploration, in macaque hippocampus. However, the association between SWRs and multiple forms of awake conscious and goal-directed behavior is unknown. We report that ripple activity occurs in macaque visual areas V1 and V4 during focused spatial attention. The occurrence of ripples is modulated by stimulus characteristics, increased by attention toward the receptive field, and by the size of the attentional focus. During attention cued to the receptive field, the monkey’s reaction time in detecting behaviorally relevant events was reduced by ripples. These results show that ripple activity is not limited to hippocampal activity during offline states, rather they occur in the neocortex during active attentive states and vigilance behaviors.

## Significance

Short-lived, high-frequency episodes in the electrical activities of mammalian hippocampus, called ripples, occur during sleep and quiescence, and are linked to memory formation. To which extent they occur during active behaviors outside hippocampus is poorly understood. We detected ripples in visual areas V1 and V4 of macaque monkeys during top-down spatial attention. Ripples occurred at frequencies not dissimilar to rates reported in the hippocampus. Critically, the ripple rate was modulated by attention cued to the receptive field, by stimulus size, and by the size of the attentional focus. Moreover, reaction times were modestly reduced on trials where ripples occurred.

Hippocampal sharp-wave ripples (SWR, ripples) are large amplitude deflections (sharp-waves) of the local field potential (LFP) in the hippocampus of rodents, humans, and nonhuman primates, associated with a brief fast oscillatory pattern (ripple). Ripple oscillations vary in frequency from 140 to 200 Hz in rodent and 80 to 180 Hz in nonhuman primates and humans (1–6). SWRs occur at ~0.5 Hz in the hippocampus, 0.1 to 0.5 Hz in the posterior parietal, retrosplenial, cingulate, and at 0.05 Hz in somatosensory, motor, and visual cortices during nonrapid eye movement (NREM) sleep (7). During hippocampal SWRs, 15% of hippocampal pyramidal cells discharge synchronously, which triggers activation in cortical areas, but suppression in midbrain and brainstem regions (2, 3).

Ripples support memory consolidation by transferring information acquired during waking to cortical networks during sleep and quiescence (7–9). Consolidation occurs through temporal replay of event-related activity in the hippocampus during ripples (10–15). SWRs are also predictive of future trajectory and performance during spatial navigation tasks (16–18). Finally, they are implicated in the correct temporal sequencing of place cell activity preceding novel spatial experiences (preplay) (19). Memory consolidation in the visual cortex requires NREM sleep spindle activity (20), which are coordinated with hippocampal ripples (21), increasing hippocampal–neocortical coupling (22) and associated information transfer.

In rodents, hippocampal SWRs are pronounced during offline states (23, 24), but they occur during awake states in humans (25), as well as nonhuman primates during visual search and goal-directed visual exploration, termed exploratory SWRs (26, 27). Hippocampal SWR occurrence of monkeys is increased when the subject’s gaze is focused near a target object during search or when patients observe familiar pictures of scenes or faces (5, 26, 28). Here, ripple rates also increased during free recall along with a high-frequency band activation around the time of ripple in the visual cortex, suggesting a role of SWRs in activating the visual cortex during episodic and semantic memory retrieval (5, 19, 26, 28–31). Memory and attention are intertwined, and working memory and attention affect neural activity in striate and extrastriate cortex similarly (32, 33). Given that ripples are strongly linked to memory, it is tempting to speculate that they are linked to attention.

Attention affects components of neural circuits, that in the hippocampus drive ripples. Sharp-waves are generated by excitatory afferents from CA3 to CA1 (34, 35), but ripples are evoked by parvalbumin-positive (PV^+^) interneurons inside CA1 (36). Optogenetic activation of PV^+^ cells induces hippocampal ripples (37), and PV^+^ cells can be active during each ripple cycle (38, 39). Narrow spiking cells, often associated with PV^+^ cells (40) are more affected by spatial attention than broad spiking cells (41). Hence, we hypothesize that ripples increase in the visual cortex when animals are cued to attend to the receptive field (RF, referred to as *cue RF* conditions), relative to when animals are cued to attend to the opposite hemifield (cue away conditions), as PV^+^ drive is likely increased.

Attention might also increase ripple rates through cholinergic mechanisms. Ripples during offline states coincide with reduced septal acetylcholine (ACh) release into the hippocampus, and cholinergic suppression of hippocampal SWR impairs spatial working memory (42). ACh plays an important role in working memory and spatial attention, and hence SWRs should decrease during cue RF conditions, if ACh levels are increased. However, cholinergic receptor distribution differs between the hippocampus and primate visual cortex. In the human hippocampus, M1 receptors are predominantly expressed in excitatory pyramidal cells (43), while in the (primate) visual cortex, they are predominantly expressed on inhibitory interneurons, and here especially on PV^+^ cells (44). Hence, increased ACh levels with attention (45) might trigger higher ripple rates in the visual cortex. SWRs have been suggested to be an alternative working memory system to the proposed system of “delay activity” or “neuronal chaining” and theta activity (46, 47). If SWRs help refocus by “reminding” the system about current task demands, then we hypothesize that ripples increase when animals are required to attend to the RF under cue RF conditions.

In rodents, somatostatin-positive (SOM^+^) interneurons are suppressed during ripple episodes (39), and SOM^+^ cell activity is linked to surround suppression (48). Whether similar mechanisms occur in the primate neocortex, where SOM^+^ is not a good marker for interneurons (49, 50), is unknown. However, primate calbindin-positive (CB^+^) cells may be homolog to rodent SOM^+^ cells, and may serve a similar function. If so, spatial attention, which causes surround “exclusion” (51), might do so through reduced CB+ activity (51, 52), and thereby increase ripple rates. The link between increased SOM^+^ activity and reduced ripple rates (in rodents) further suggests that larger stimuli, inducing surround suppression and higher SOM^+^ (CB^+^) cell activity, result in reduced ripple rates.

To examine these predictions, we recorded LFPs and spiking activity in visual areas V1 and V4 of two male macaque monkeys performing a cued spatial attention task. Ripple activity was detected in both regions, ripples occurred more often when monkeys were cued to deploy attention to the RF of the recorded neurons, smaller stimuli resulted in higher ripple rates than larger stimuli, and ripple occurrence was predictive of better behavioral performance. Thus, ripples occur in cortical visual areas and are involved in cognitive functions beyond memory consolidation and retrieval.

## Results

**Fig. 1.**
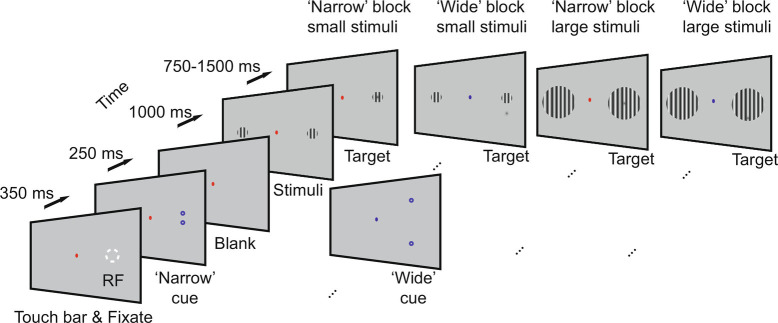
Behavioral paradigm. Monkeys touched a touch bar, which triggered FP appearance on the screen. Following fixation onset, two cues (small annuli) were presented for 250 ms, indicating which side of the screen was behaviorally relevant. The distance of the two rings, as well as the color of the fixation spot, indicated whether the trial belonged to a narrow or wide focus of attention condition; 1,000 ms after cue offset, two static grating were presented, one centered on the RF (white dotted circle, for illustrative purpose, not present on the screen during the experiment), the other grating appeared equidistant to the fixation spot in the opposite visual hemifield. After 750 to 1,500 ms a dimming spot appeared in the cued location, upon which the monkey had to release the touch bar within 500 ms to receive the fluid reward. In narrow blocks the dimming spot was generally presented centered on the target stimulus while in wide blocks, it was generally presented at an unpredictable location offset from stimulus center.

Monkeys performed a covert spatial attention paradigm (*Materials and Methods*, Fig. 1). We recorded 26 and 17 sessions in monkeys 1 and 2, respectively, with overlapping RFs between V1 and V4 (*SI Appendix*, Table S1). We analyzed three main epochs for ripple occurrence, namely i) precue: a 200-ms period after fixation before cue onset, ii) postcue (800-ms period after cue offset until stimulus onset), and iii) the sustained response period corresponding to 300 to 750-ms after stimulus onset (*SI Appendix*, Fig. S1*B*).

### Ripple Attributes Across Task Epochs.

**Fig. 2.**
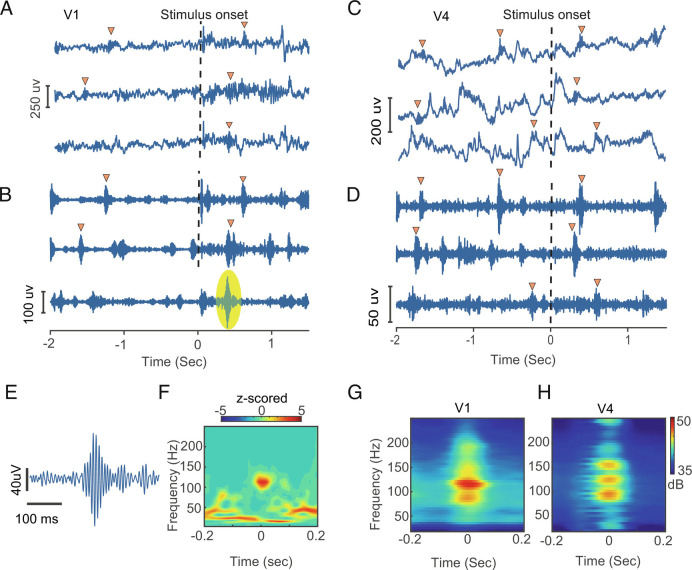
Example ripples recorded during task epochs time locked to stimulus onset. *A *and *C*) Bipolar rereferenced LFPs demonstrating occurrence of ripples in the task epochs in V1 and V4. *B* and *D*) respective ripple band voltage traces (bandpass filtered 80 to 250 Hz). Time zero and dotted line indicate stimulus onset. Ripples are shown with yellow triangles. *E* and *F*) Representative magnified ripple trace and spectrogram indicated with a yellow oval in *B*. Color bar represents z-scored power. *G* and *H*) Overall average of Hanning windowed taper spectrogram showing time–frequency distribution of ripples identified in V1 (*G*) and V4 (*H*). Color maps reflect power of ripples in decibel (dB).

In V1, we detected n = 166, 688, 7,768 ripples for precue, postcue, and sustained periods, respectively. In V4, the numbers were n = 642, 2,740, and 9,545. Given the variable duration of the periods used, these numbers are not comparable. We thus convert ripple rates to events per second (ES) for the remainder of the manuscript. Fig. 2 shows example trials with ripples during the task epochs. Spectrograms of ripples revealed a high-frequency oscillation (100 to 180 Hz) in V1 as well as in V4 (Fig. 2 and *SI Appendix*, Figs. S2 and S3). Ripples in V1 showed a lower peak frequency during the sustained compared with pre and postcue periods, while in V4 ripples had a higher peak frequency during the sustained period [*SI Appendix*, Fig. S3, V1; χ_(2)^2^_ = 15.9365, *P* < 0.001, V4; χ_(2)^2^_ = 145.7693, *P* < 0.001, Kruskal–Wallis (KW) test]. The majority of ripples had durations between 30 and 60 ms. On average, the mean and SEM of durations of ripples were 45.19 ± 1 ms, 43.95 ± 0.5 ms, and 47.67 ± 0.5 ms in V1 for precue, postcue, and sustained periods, respectively. In V4, these durations were 47.6 ± 1 ms, 45.2 ± 0.5 ms, and 47.8 ± 1 ms (*SI Appendix*, Fig. S4). Ripples during the sustained period were longer than in other periods in both areas (V1; χ_(2)^2^_ = 26.8762, *P* < 0.001, V4; χ_(2)^2^_ = 59.7597, *P* < 0.001, KW test).

To ensure that ripples were not a consequence of spike contamination of the LFP signal, we applied a Bayesian spike removal algorithm (53) on three randomly selected sessions of the data. Removing contamination of spikes local to the electrode also allows differentiating a very local phenomenon from more widespread oscillatory signals. First, we calculated the spike-triggered average (STA) between manually sorted spikes (*Materials and Methods*) and the LFP for each channel. Spike leakage into the LFP occurred on some contacts (*SI Appendix*, Fig. S5). Following spike removal, STA was computed again and compared with the original STA. Spike leakage was removed by the Bayesian estimate (*SI Appendix*, Fig. S5). Critically, the ripple rate was not reduced by spike removal (*SI Appendix*, Fig. S6).

### Ripples and Cortical Layers.

We analyzed whether ripple rates differed between cortical layers. The depth of recording contacts was determined by multiunit response latency and current source density (CSD) analysis of LFPs (*Materials and Methods*). *SI Appendix*, Fig. S7 illustrates the CSD profile and laminar alignment to characterize layers. Layers that showed the fastest response latency and the early current sink were labeled as “input layer.” Layers located above the input layer (presumably layers I–III) were labeled superficial, and layers below the input layer (presumably V and VI) were labeled deep layers. As the thickness of layers varies within and between visual areas, we divided ripple rates over the number of the contacts embedded in the corresponding layer. Ripple rates were similar in superficial, input, and deep layers of V4 (F(2,126) = 0.57, *P* = 0.56, one-way ANOVA). In V1, the input layer showed higher ripple rates than superficial and deep layers (one-way ANOVA over pooled data and layer as factors, F(2,119) = 9.44, *P* < 0.001, post hoc Tukey: superficial vs. input, mean difference = −0.04, 95% CI = [−0.06, −0.01], *P* = 0.002, input vs. deep: mean difference = 0.04 CI = [0.01, 0.07], *P* = 0.003). The ripple rate did not differ between V1 superficial and deep layers (*SI Appendix*, Fig. S8).

### Effect of Stimulus Size on Ripple Events.

**Fig. 3.**
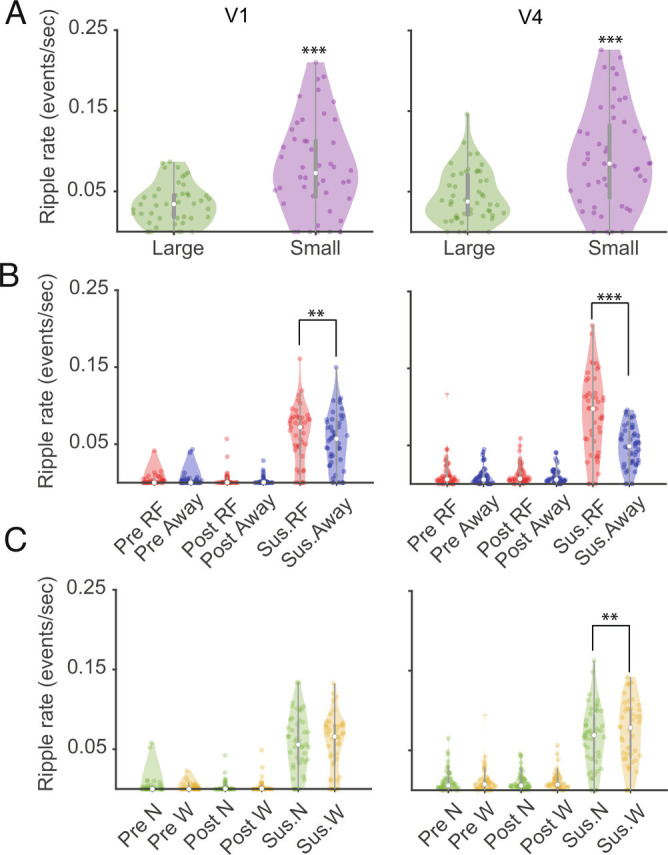
Ripple rate as a function of stimulus size, attentional cueing and size of focus of attention. *A*) Effect of stimulus size on ripple rates. *Left* column shows the ripple rate in V1 for small and large stimuli. *Right* column shows the ripple rate as a function of stimulus size for V4. *B*) Comparison of ripple rates for different attention conditions (cue RF vs. away) for three different trial periods (precue, postcue, and poststimulus sustained period). *Left* column shows V1 data (red—cue RF, blue—cue away). *Right* column shows data for V4. *C*). Ripple rate as a function of size of attentional focus (narrow vs. wide) for the three trial periods (precue, postcue, and poststimulus sustained period). Ripple rates did not differ between attentional focus size in V1 (*Left*) for either monkey. In V4, wide focus of attention triggered higher ripple rates than narrow focus of attention. N and W indicate narrow and wide blocks of attention. The white circle indicates median, and gray rectangle represents first and third quartiles of the data. Data are presented as mean ± SEM across recordings; ** and *** indicate significant levels of *P* < 0.01 and *P* < 0.001, respectively.

Small stimuli were associated with higher ripple rates than large stimuli in both areas (consistent across subjects). Mean and SEM of ripple occurrence rates in V1 and V4 for small stimuli were 0.09 ± 0.006 ES and 0.1 ± 0.007 ES, while they were 0.04 ± 0.002 and 0.04 ± 0.003 ES for large stimuli (V1: Z = −4.7, *P* < 0.001; V4: Z = −4.4, *P* < 0.001, Wilcoxon signed rank test, Fig. 3*A*). The effects of stimulus size on the ripple rate were not an artifact of centering the stimuli on the (smaller) V1 RFs, potentially causing offsets between V1 and V4 RF stimulation, and possibly not stimulating some V4 RFs when small stimuli were used. First, if that was the case, lower ripples rates, comparable to prestimulus periods for small stimuli in V4 would occur, not higher ripple rates. Secondly, in monkey 1, most recordings had V1 RFs that were completely covered by all V4 RFs, i.e., the small stimuli covered both RF locations well (*SI Appendix*, Fig. S9). For these recordings (n = 18), we found the same effect of stimulus size on ripple rates as for the entire set of recordings (*SI Appendix*, Fig. S10; additional details see: *SI Appendix, SI Ripple Rate and RF Overlap*). The dependence on stimulus size suggests that the ripple rate might also be related to RF size and eccentricity, but here only small effects were found for V4 RF size (*SI Appendix*, Fig. S11).

### Relation of Ripples to Attention States, and Possible Interactions with Stimulus Properties.

We analyzed ripples across three task epochs. The mean and SEM of ripple rates in V1 was 0.0031 ± 0.009 and 0.0033 ± 0.008 ES for pre- and postcue, and 0.056 ± 0.002 ES during sustained periods. In V4, the mean and SEM of ripple rates was 0.01 ± 0.001 ES during pre- and postcue and 0.068 ± 0.003 ES during the sustained period. Ripple rate in intertrial intervals was 0.02 ± 0.002 ES for both regions.

We next analyzed whether attention influenced ripple rate. Ripple rate did not differ between cue RF and cue away during pre- and postcue periods in V1 (precue: Z = 1.06, *P* = 0.28, postcue: Z = 0.36, *P* = 0.71, Wilcoxon test) or V4 (precue: Z = 0.95, *P* = 0.33, postcue: Z = 1.36, *P* = 0.08, Wilcoxon test). However, during the sustained period the mean and SEM of ripple rates was higher for cue RF conditions in V1 (0.06 ES in cue RF vs. 0.05 ES in cue away, Wilcoxon sign rank test; V1: Z = 2.4732, *P* = 0.0134, Fig. 3*B*), and also in V4 (0.09 for cue RF vs. 0.04 for cue away, Wilcoxon sign rank test; Z = 5.66, *P* < 0.001, Fig. 3*B*). Fig. 3 shows the overall distribution of ripple rates for the effects of stimulus size, cued locus of attention, and focus of attention across sessions.

The above analysis did not dissect effects according to different stimulus and attentional focus conditions. To assess whether effects of cued attention depended on stimulus size and/or the size of the attentional focus, we calculated ripple rates for all triplet-wise conditions (eight possible permutations: attend(cue)-RF/small-stimuli/narrow-focus, attend(cue)-RF/small-stimuli/wide-focus, attend(cue)-RF/large-stimuli/narrow-focus, …) for each electrode for each session, and then averaged ripple rates across electrodes for a given session. This yielded eight values corresponding to the eight possible condition combinations for each session. We then used a three-factor repeated measure (RM) ANOVA, to determine main effects of stimuli, cued attentional location, and attentional focus (see *Materials and Methods* and *SI Appendix*, Fig. S15 regarding the efficacy of manipulating the animal's size of attentional focus), and possible interactions between these factors on ripple rates in V1 and V4.

#### Effects in area V1.

**Fig. 4.**
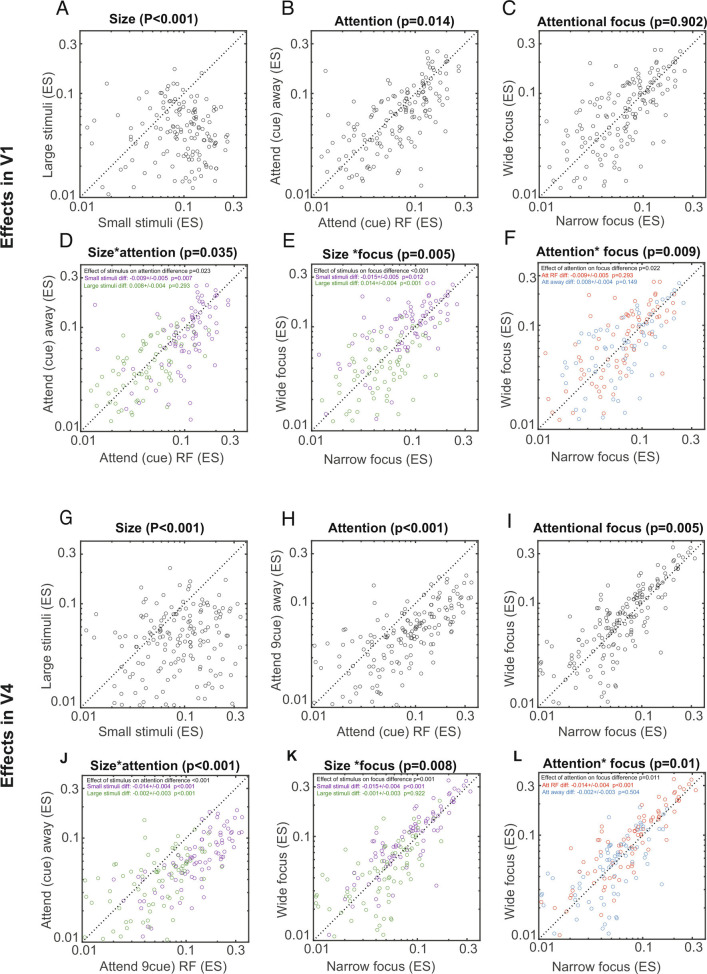
Ripple rate in areas V1 and V4 as a function of stimulus size, attentional cueing, and attentional focus, as well as possible pairwise interactions. *A*) Main effect of stimulus size on ripple rate. *B*) Main effect of attentional cueing (location) on ripple rate. *C*) Main effect of focus of attention on ripple rate. *D*) Interaction between stimulus size and attentional cueing location on ripple rate. Purple: ripple rate for small stimuli for cue RF vs. cue away conditions. Green: ripple rate for large stimuli for cue RF vs. cue away conditions. *E*) Interaction between stimulus size and focus of attention on ripple rate. Purple: ripple rate for small stimuli for narrow focus vs. wide focus conditions. Green: ripple rate for large stimuli for narrow focus vs. wide focus conditions. *F*) Interaction between attentional cueing locus and focus of attention on ripple rate. Purple: ripple rate for cue RF conditions for narrow focus vs. wide attentional focus. *G*–*L*) Same as *A*–*F* but for area V4. Purple: ripple rate for cue away conditions for narrow focus vs. wide attentional focus. Text insets in each panel provide FDR corrected *P*-values from post hoc sign rank tests, along with means ± SEM for each color-coded population. Non colored text *Insets* are between group comparisons (e.g., a given green vs. purple comparison, FDR corrected post hoc sign rank test *P*-values).

The three-way RM-ANOVA revealed that stimulus size (*P* < 0.001, Fig. 4*A*) and attention (cue RF vs. away, *P* = 0.014, Fig. 4*B*) had a main effect on ripple occurrence in V1 data, while the focus of attention did not have a main effect (*P* = 0.902, Fig. 4*C*). Details about the statistics (df, F-values, …) see *SI Appendix*, Table S2. Small stimuli resulted in higher ripple rates than large stimuli, and attention cued to the RF resulted in higher ripple rates. Additionally, we found a significant stimulus*attention (location) interaction (*P* = 0.035, Fig. 4*D*), a significant stimulus*attentional focus interaction (*P* = 0.005, Fig. 4*E*), and a significant attentional location*focus interaction (*P* = 0.009, Fig. 4*F*). The effect of cued attentional location on ripple rate was significant when small stimuli were presented [*P* = 0.007, post hoc sign rank test, false discovery rate (FDR) adjusted *P*-value, Fig. 4*D*], but not when large stimuli were presented (*P* = 0.293 FDR adjusted, post hoc sign rank test, Fig. 4*D*). For the stimulus*attentional focus interaction, we found that wide foci of attention yielded higher ripple rates than narrow foci when small stimuli were presented (*P* = 0.012, FDR adjusted, post hoc sign rank test, Fig. 4*E*). The opposite was the case when large stimuli were presented (*P* < 0.001 FDR adjusted, post hoc sign rank test, Fig. 4*E*). This difference itself was significant (*P* < 0.001 FDR adjusted, sign rank test based on pairwise differences for the two groups, Fig. 4*E*). Finally, the locus of attention*focus of attention interaction was a result of slightly increased ripple rates for wide focus of attention under cue RF conditions, and slightly decreased ripple rates for wide focus of attention conditions under cue away conditions (always compared with narrow/small focus). However, neither of these two comparisons were significant by themselves (FDR adjusted post hoc sign rank test, details in Fig. 4*F*).

#### Effects in area V4.

The effects of stimulus size, cued attention location and attentional focus, and possible interactions for area V4 are shown in Fig. 4 *G–L*. The three-way ANOVA revealed that stimulus size (*P* < 0.001, Fig. 4*G*), attentional cueing (*P* < 0.001, Fig. 4*H*), and attentional focus (*P* = 0.005, Fig. 4*I*) had a main effect on ripple frequency in V4 (statistical details are listed in *SI Appendix*, Table S3). Small stimuli resulted in higher ripple rates than large stimuli. Cued attention to the RF (compared with cue away) resulted in higher ripple rates, and a wide focus of attention (compared with narrow focus) resulted in higher ripple rates. Additionally, we found a significant stimulus*attentional location interaction (*P* < 0.001), a significant stimulus*attentional focus interaction (*P* < 0.001), and a significant attentional location*attentional focus interaction (*P* = 0.005). Cueing attention to the RF resulted in higher ripple rates than cueing away, when small stimuli were presented (*P* < 0.001 FDR adjusted, sign rank test, Fig. 4*J*). While this was also the case when large stimuli were presented (*P* < 0.001 FDR adjusted, sign rank test, Fig. 4*J*), the effect of attentional cueing on ripple rates was significantly stronger when small stimuli were presented than when large stimuli were presented (sign rank test on pairwise differences between cue RF minus cue away conditions, grouping variable: small vs. large stimuli, *P* < 0.001 FDR adjusted, Fig. 4*J*). A wide focus of attention resulted in higher ripple rates than a narrow focus of attention, when small stimuli were presented (post hoc sign rank test, *P* < 0.001 FDR adjusted, Fig. 4*K*), but not when large stimuli were presented (post hoc sign rank test, *P* = 0.922 FDR adjusted, Fig. 4*K*). This difference itself was significant (*P* = 0.001 FDR adjusted, sign rank test on differences between narrow and wide foci of attention for small vs. large stimuli). Finally, when attention was cued to the RF a wide focus resulted in higher ripple rates than a narrow focus (*P* < 0.001 FDR adjusted, post hoc sign rank test, Fig. 4*L*), while no difference for the focus of attention was found when attention was cued away from the RF (*P* = 0.504 FDR adjusted, post hoc sign rank test, Fig. 4*L*). The difference of cue RF vs. away on attentional focus difference was significant (*P* = 0.011 FDR adjusted, sign rank test, Fig. 4*L*).

### Spiking Activity at Ripple Time.

**Fig. 5.**
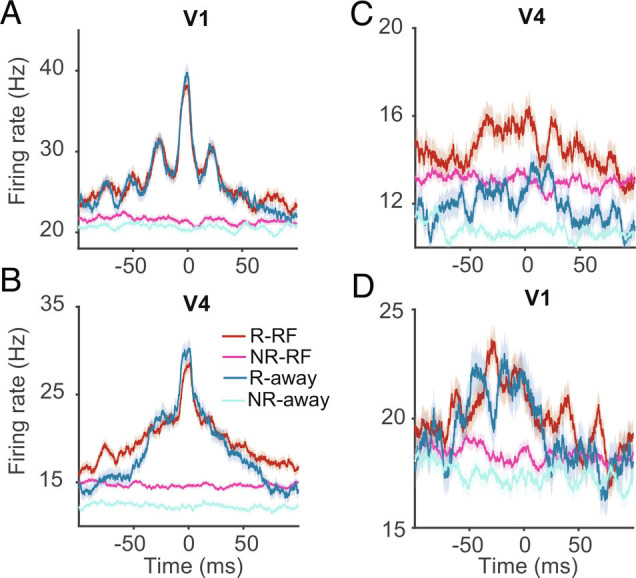
Spiking activity with and without ripple events. Mean and SEM of spiking activity centered at ripple time in V1 (*A*: V1 spiking activity-V1 ripples; *D*: V1 spiking activity-V4 ripples) and V4 (*B*: V4 spiking activity-V4 ripples; *C*: V4 spiking activity-V1 ripples) on trials with and without ripple. Dark red and blue lines show trials with ripples on cue RF/away conditions, pink and cyan are trials without ripples, chosen from the same number of trials as with ripples, under identical conditions (i.e., cue RF/away, narrow/wide focus, …), and matched time points within a trial. For trials without ripples the analysis was repeated 10 times with random selection of matched trials (with replacement) to control for robustness of difference (activity shown is averaged across the 10 repetition). R-RF, and R-away: ripples during attentional cueing to RF and away conditions. NR-RF and NR-away: no ripples during attentional cueing to RF and away conditions.

We next compared neural firing rates (*Materials and Methods*) during the sustained period at ripple time to firing rates during trials without a ripple for identical conditions (Fig. 5 and *SI Appendix*, Tables S4 and S5 give a breakdown of firing rates for the multiple conditions tested in V1). We did this for firing rates within an area, when a ripple occurred in that area (Fig. 5*A* for V1 and 5*B* for V4), compared with rates within that area, without a ripple in the area. We also investigated firing rates in an area, when a ripple occurred in the “other” area, but not in the area where we investigate the firing rates (Fig. 5*C* for rates in V4 when ripples occurred in V1 and Fig. 5*D* for rates in V1 when ripples occurred in V4). In V1, with a time window of ±100 ms around ripple time, the mean and SEM of firing rate during the sustained period without ripples and cue RF trials was 21.8 ± 0.2 Hz, which increased to 29.6 ± 0.3 Hz in trials with ripples. In cue away trials, ripples were associated with enhanced average firing rates, which attained 29. 3 ± 0.3 Hz, while in the same condition without ripples the rate was 21.5 ± 0.2 Hz. Similarly, in V4, the mean and SEM of firing rate during the sustained period in trials without ripples and cue RF was 15.5 ± 0.1 Hz, which increased to 24.0 ± 0.2 Hz with ripples. Ripples in cue away trials were associated with mean firing rates of 23.1 ± 0.3 Hz, while without ripples, rates were 13.1 ± 0.1 Hz. Fig. 5 also gives an overview of firing rates in trials with and without ripples for condition and trial-matched data for V1 and V4 (random selection of trials, repeated 10 times and averaged over those repetitions). To further explore which factors of the task contribute to increased neural activity, we conducted a linear mixed-effect model analysis to predict the firing rate based on attention (cued RF/away), ripple occurrence (presence/absence), focus of attention (narrow/wide) and size of stimulus (large/small). The mixed effects model in V1 revealed that the firing rate was modulated by ripple, focus of attention, and stimulus size [ANOVA following mixed-effect model: Ripple: F(1, 15,037) = 28.2, *P* < 0.001, size: F(2, 15,037) = 211.6, *P* < 0.001, ripple*size: F(2, 15,037) = 3.8, *P* = 0.02]. Similarly, in V4 firing rate was modulated by ripple, attention to RF and size of the stimulus. (ANOVA following model: Ripple: F(1, 14,356) = 11.1, *P* < 0.001, size: F(2, 14,356) = 28.1, *P* < 0.001, Attention*size: F(2, 14,356) = 8.2, *P* < 0.001, Focus*size: F(2, 14,356) = 5.1, *P* = 0.005). *SI Appendix*, Tables S6 and S7 summarize the results of the linear mixed-effect model in V4.

Thus, firing rates peak around the time of a ripple. This was pronounced if the ripple occurred within the same area. activity was also increased in an area, when ripples occurred in remote areas. For this scenario, area differences appear in terms of timings of effects. A ripple in V1 was accompanied with elevated firing rates in V4, with a very gentle “hill” centered on the V1 ripple time (Fig. 5*C*). Conversely, a ripple in V4 was associated with elevated firing rates in V1, which appeared to peak before the V4 ripple center, and drop at the time of the V4 ripple center, possibly with a rebound after the V4 ripple for cue RF conditions (Fig. 5*D*). This suggests that elevated activity in V1 might trigger V4 ripples.

### Cross-Correlation of V1 to V4 Ripples.

**Fig. 6.**
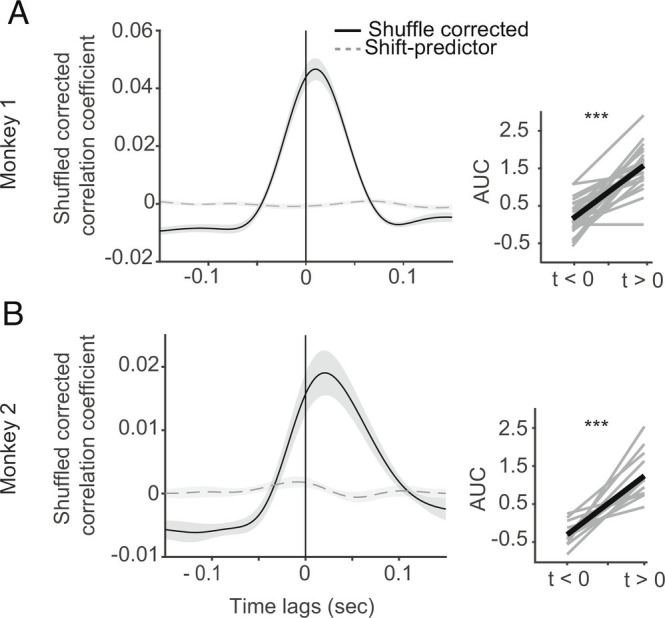
Temporal cross-correlation between ripples in V1 and V4. *A*) Black curve in the *Left* panel represents shuffle corrected correlation of V1 and V4 ripples in monkey 1. The dashed gray curve corresponds to cross-correlation of shuffled corrected trials. *Inset* in the *Right* panel indicates area under the curve for lead (AUC to left of ripple center) and lag AUC. *B*) Same as panel *A* but for monkey 2. Data are presented as mean ± SEM across recordings; *** indicates significant level of *P* < 0.001.

To assess this further, we computed cross-correlations between V1 and V4 ripples between all channel combinations, pooled across session (Fig. 6). Cross-correlations were shuffle predictor corrected. Cooccurrence probability of ripples on any contact in V1 and V4 during the sustained period for a given trial was 0.1 (relative to all ripples in just one area). The area under the cross correlogram (AUC) relative to time zero was used to investigate lead-lag relationships. AUC values were significantly skewed to positive values (monkey 1: Z = −4.3724, *P* = 1.2290e−05, monkey 2: *P* = 2.4414e−04, Wilcoxon signed rank test), and the cross-correlation exhibited a peak at approximately 20 to 25 ms, indicating that V1 ripples on average occurred before V4 ripples.

### Cross-Frequency Power Coupling between Ripples.

**Fig. 7.**
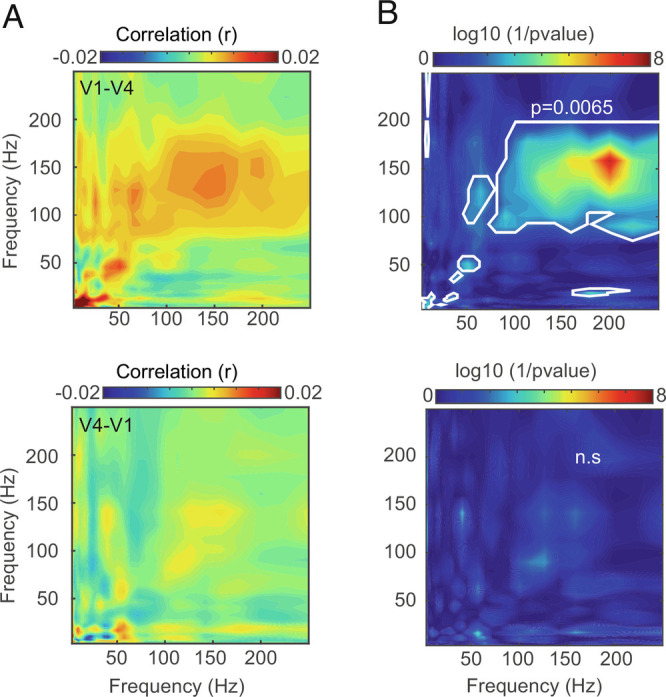
Modulation of spectral power between V1 and V4 LFPs around the time of ripple events. *A*) *Upper* panel shows average comodulogram of V1 and V4 when V1’s ripples served as the trigger. Comodulogram demonstrated frequency coupling between V1 and V4 at the ripple and gamma frequency band. X and y axes correspond to V1 and V4 frequencies, respectively. *Lower* panel represents average corrected comodulogram of frequency coupling between V4 and V1 where ripples in V4 served as trigger. Here, x axis correspond to V4 frequency and y axis indicates V1 frequency. *B*) Logarithm with base 10 of 1/*P*-values after FDR correction for V1 (*Upper*) and V4 (*Lower*). Points inside solid bright line represent frequency bands that showed significant coupling at V1’s ripple episodes (FDR corrected).

Is ripple activity coordinated between layers, between areas, and is there cross frequency coupling to other frequency bands? To examine this, we estimated the frequency-dependent spectral power correlation of V1 and V4 LFPs using either V1 or V4 ripples as trigger events. Fig. 7 depicts the average of power coupling across channel combinations of V1 and V4 using V1 ripples as the trigger. Power–power coupling was dominant for the ripple and gamma band between V1 and V4 (at α = 0.05, *P* = 0.0065, FDR correction). However, power correlation was not significant between areas when V4 ripples served as trigger. This suggests that ripples in V1 affect LFP spectral power in V4, i.e., in the feedforward, but not the feedback direction.

To determine layer-specific coordination, we analyzed the power correlation between pairs of contacts in the three laminar compartments within each area, using ripple events as triggers as described above. The comodulogram profile for V1 showed that the power–power coupling between superficial to superficial, input to superficial, and deep to superficial layers was higher in the ripple band. This effect was pronounced in the reverse direction as well (*SI Appendix*, Fig. S12*A*). In V4, the power coupling was significant when deep layer contacts served as a trigger for the superficial contacts, as well as superficial to superficial contacts, with no other significant interactions (*SI Appendix*, Fig. S12*B*).

### Ripple Rate and Reaction Time (RT).

We computed RTs in different task conditions across all trials to determine whether it was correlated with ripple occurrence. Regardless of task conditions, the mean and SEM of RTs on trials with ripples was 308.49 ± 0.5 ms, in trials without ripples, it was 310.9± 0.5. This difference was significant, i.e., subjects were faster to detect the target on trials with ripples [t(1, 14,970) = −3.2, *P* = 0.001, two-sided *t* test]. Next, RTs were classified by no ripple, ripple occurrence in V1, ripple occurrence in V4, and finally ripples in both areas. Mean and SEM of RTs on trials without ripples was 310.9 ± 0.5 ms, whereas on trials when ripples only occurred in V1, mean and SEM were 310.9 ± 0.8 ms. RT in trials where ripples only occurred in V4 was 307.5 ± 0.8 ms. When ripples occurred in V1 and V4, mean and SEM of RT was 305.5 ± 1.13 ms (*SI Appendix*, Fig. S13). To probe this further, a linear mixed-effect model was used to identify which task variables were significant predictors of RT. Attention cued to/away from RF, focus of attention (wide–narrow), stimulus size, and ripple occurrence (no ripple, V1 or V4 ripple, ripple on both areas), were used as predictors. Ripples reduced the RT of the monkeys [F(3, 14,970) = 5.04, *P* = 0.001, ANOVA following linear model]. Attention (cue) to RF and stimulus size also decreased RTs [Attention: F(1, 14,970) = 10.9, *P* < 0.001, size: F(1, 14,970) = 10.8, *P* = 0.001, ANOVA following model]. This suggests that ripple occurrence affects the RT of the subjects. *SI Appendix*, Tables S8 and S9 give details of the linear model on RTs.

## Discussion

Our results reveal new aspects of ripples and their association with visual attention. First, the ripple rate was higher when small stimuli rather than large stimuli were presented. Second, top-down attention cued to the RF enhanced the ripple rate in V1 and V4. Ripples were associated with higher activity not only in the area where the ripple occurred, but also in the “remote” area, if no ripple occurred in that area. When ripples jointly occurred in both areas, V1 ripples generally preceded V4 ripples, and a spectral power correlation in the ripple band between striate and extrastriate area V4 was significant in the feedforward, not the feedback direction.

SWRs occur most frequently during quiescence and NREM sleep in the hippocampus of rodents where they are important for memory consolidation (10, 12, 54) and less frequent during waking periods where they are important for memory-guided decision-making (55–57), or maze navigation where they predict future navigational behavior (17, 58–60). When rodents learn a navigation task, SWR rate increased before successful trials during the learning period (18). SWRs have recently been reported in the primate hippocampus, where they occur during active visual search and memory retrieval (26, 28) suggesting they are required to integrate immediate past experiences with current rules (61). Most previous studies have reported SWRs in the hippocampus, but they have recently also been found in the rodent association cortex (7) during NREM sleep and quiet wakefulness. In humans, increased ripple rates occur in the hippocampus during autobiographical episodic and semantic memory recollection tasks (5, 30). Our study shows the modulation of ripple rates by spatial attention cueing, and by visual stimulus characteristics in the striate and extrastriate areas in the nonhuman primate, during a cued covert spatial attention task. Critically, attentional cueing to the RFs of the neurons increased ripple rates and ripple occurrence resulted in faster RTs. Many studies implicate SWRs in memory retrieval and planning. A possible interpretation of ripples in the visual cortex during spatial attention is that the subjects had to memorize the location of a spatial cue to detect the target. Thus, ripples in our study might indicate a form of memory recollection to accomplish the task. However, memory in our task would probably be working memory, rather than spatial (or even episodic) memory as probed in previous studies. Studies in rodents uncovered that ripples are prevalent at reward sites of a navigation task (62). In our paradigm, attention was linked to the likelihood of receiving a reward, which may explain the higher ripple rate during the sustained period with cue RF conditions. A higher ripple rate with wide attention is also consistent with this, because the uncertainty of the subjects was higher, therefore they may demand more attention to receive a reward. A wide focus of attention also has a covert search component, and may thus be linked to increased ripple rates seen in the primate hippocampus during active visual search (26, 28, 63). However, all these suggestions are currently speculation, and further studies are required to investigate this.

Which mechanisms might trigger the increased ripple rates with attentional cueing to the RF and when small, compared with large stimuli are presented? Hippocampal ripples are evoked by PV^+^ interneurons (36, 37). Attention in the visual cortex has a larger effect on narrow spiking cells (40), which are often argued to be predominantly PV^+^ cells (but see refs. 41, 64, and 65). Hence if attention increases PV^+^ cell activity, it might lead to increased ripple rates. Attention has also been linked to “surround–exclusion” mechanisms (51, 52), possibly reducing surround suppression. Surround suppression is partly mediated through SOM^+^ cells in rodents (48), which show reduced activity during hippocampal ripples (39). If we infer that reduced SOM^+^ activity promotes higher ripple rates, then this might be another mechanism by which attention to the RF increases ripple rates, particularly with wide foci of attention. Conversely, larger SOM^+^ cell activity, as induced by increased surround suppression with large stimuli (66) would result in reduced ripple rates, as also seen in our data (but see the note of caution in the introduction about rodent SOM^+^ cell and primate CB^+^ cell equivalence). It might be argued that there should be discrepancies between the effects seen in V1 and V4, as cells in V4 have larger RFs than V1 cells. Thus, the larger stimulus might not extend into the suppressive surround of V4 cells, while it does for V1 cells. However, in our data larger stimuli elicit surround suppression in both areas (*SI Appendix*, Fig. S14).

There is a wealth of research indicating high-frequency oscillations are involved in the feedforward and intracortical communication (67–70), Ripples in V1 and V4 could also serve this function, possibly maintaining specific communication subspaces (71) required for efficient task performance. The fact that cortical ripple band activity in V1 was coupled with V4 gamma, that V1 activity was elevated before V4 ripples, and that V1 ripples preceded ripples in V4 might be a signature of this. Future work is required to explore these possibilities.

## Materials and Methods

### Animals and Procedures.

All surgical and behavioral procedures conformed to the UK Animals Scientific Procedures Act, European Communities Council Directive RL 2010/63/EC and the US NIH Guidelines for the Care and Use of Animals for Experimental Procedures.

Two adult male rhesus monkeys (10 and 12 kg, aged 8 and 9 y) were trained to comfortably sit in a plexiglass chair. Before training, they had been implanted with a head holder device under sterile conditions. Two recording chambers were implanted over areas V1 and V4 under sterile conditions. Details of surgical procedures, postsurgical management and analgesics have been described previously (72). To promote behavioral motivation and performance, daily access to fluid was controlled during training and experimental periods, using fluid control regimes with minimal psychological impact and no measurable physiological impact on the animals (73).

### Visuospatial Attention Task.

Monkeys detected a small, low contrast luminance stimulus appearing on a square wave grating in the peripheral visual field while centrally fixating (Fig. 1). If the small stimulus occurred at a cued location, it is referred to as "target," if it occurred at an uncued location, it is referred to as "distractor." The paradigm involved cued spatial attention, whereby the cue also indicated whether the attentional focus should best be narrow or wide (details below). We refer to trials where the animal was cued to attend to the RF as "cued RF" conditions, and trials where it was cued to attend to the opposite hemifield as "cued away." Monkeys initiated a trial by holding a touch bar. Subsequently a fixation point (FP, red annulus for cue RF, blue for cue away conditions, 0.17° diameter) appeared at the center of the screen on a gray background. A drift in the eye position of more than 1.5 or 0.9° (monkey 2 or monkey 1) throughout the trial registered as a fixation error terminating the trial. Following initial fixation (monkey 2:500 ms, monkey 1:350 ms), a spatial cue (a pair of blue annuli, 0.17° diameter) was presented for 250 ms. The spatial cues served two purposes: 1) they indicated in which visual field, the target stimulus would later appear, and hence which visual location should be attended to (contralateral vs. ipsilateral to the recording locations, i.e., attend to the RF of recorded neurons vs. away), and 2) whether the best strategy would be to employ a narrow or wide attentional focus. Cue dots placed close together (cue center distance: 0.2°) indicated that the target was likely to appear centered on the stimulus (see below), while cue dots placed further apart (cue center distance: 5.0°) indicated that the target likely appeared at 1 of 12 (20 for monkey 2) possible positions peripheral to the stimulus center. One second after cue offset, two static circular square wave grating stimuli of either 1° or 5° diameter (duty cycle 1.5 cycle/degree, contrast 30%, monkey 2, or 40%, monkey 1) were presented. The grating orientation was chosen from one of six possible orientations (60° orientation spacing) to match the aggregate preference of neurons at the recording site (the orientation was fixed for a given recording session). One of the gratings was presented on the aggregate visual RF, with a focus on centering it on V1 RFs, as these were smaller. The other grating was presented in the opposite visual hemifield, mirrored symmetrical across the fixation spot. After a randomized period of 0.75 to 1.0 s or 0.75 to 1.25 s (monkey 2 and monkey 1, respectively) either the target appeared at the cued stimulus location, or the distractor appeared at the stimulus location in the opposite visual hemifield. The monkey had 500 ms to report (touch bar release) target appearance at the cued location to receive a reward (the amount of reward increased with decreasing RT) and ignore distractor appearance. If the distractor appeared at the uncued location, a target appeared at the cued location 1 to 1.4 s (monkey 2) or 1.5 to 1.75 s (monkey 1) after the distractor had appeared. The target/distractor onset latency distribution was uniform during either of these periods. The target/distractor was a circular patch of Gaussian modulated (σ = 0.1°) luminance of variable contrast relative to the parts surrounding it (which could be either the background or grating stimulus, dependent on target location and grating stimulus size). The position of the target (central/peripheral to grating center) was varied block wise. The size of the grating (large/small) was varied block wise in monkey 2, and trial wise in monkey 1. The order of target appearance (in cued location first/second) was varied pseudorandomly trial wise. Narrow and wide foci of attention blocks were additionally coded by the color of the fixation spot (narrow = red, wide = blue). For wide focus of attention conditions, the possible target position was one of at least 12 possible target positions peripheral to the grating center. In a “narrow” block, the target appeared in the center of the grating in 80% of the trials. In a “wide” block, the target appeared peripheral to the center of the grating in 80% of the trials. In invalidly cued catch trials (7% in monkey 2, 10% in monkey 1), we presented a peripheral target in narrow blocks and a center target in wide blocks. In blank trials (13% in monkey 2, 10% in monkey 1) no target was shown, and the monkey received a reward for holding on to the touch bar. RTs of monkeys were calculated by subtracting the time of touch bar release from the time of appearance of the target. *SI Appendix*, Fig. S.14 shows RT analyses associated with narrow and wide foci of attention conditions. Monkey 1 shows RTs in line with adopting narrow and wide foci strategies at least to some extent, while monkey 2 shows RTs fully in line with adopting narrow and wide foci of attention. Details about RF mapping are given in *SI Appendix*.

### Data Acquisition.

Neurophysiological recordings were performed with 16 contact laminar electrodes, 150-µm spacing between adjacent channels (monkey 1: Atlas Neuroengineering; monkey 2: V-probes, Plexon). Contact impedance was measured before each penetration (range of 0.5 to 1.0 MΩ). The probes were mounted on a hydraulic Microdrive (Narishige MO-97A). Probes were inserted perpendicular to the cortical surface intending to yield coverage of all cortical layers.

Extracellular voltage fluctuation of V1 and V4 were acquired using a 32-channel digital Lynx (Neuralynx). The signal of each electrode was referenced to a contact placed on the surface of the granulation tissue of either V1 or V4 chambers. Electrodes were connected to the recording system via a preamplifier (HS-36, Neuralynx). The raw signal was collected with 24-bit resolution, sampled at 32.756 kHz. To obtain the LFP, the raw signal was band-pass filtered offline at 0.5 to 300 Hz and down sampled to 1,017 Hz.

### Bipolar Rereferencing.

Bipolar rereferencing was performed to remove the common signal on adjacent contacts and yield a local LFP. The LFP signal was rereferenced by subtracting the signal recorded at two neighboring channels. Specifically, the signal on channel *i* was acquired by obtaining the difference signal of channel *i *+ 1 and channel *i −* 1.

$$y_{i}\left[n\right]={x_{i}}_{+1}\left[n\right]-{x_{i}}_{-1}[n],$$

where *y_i_* [*n*] denotes the signal of the rereferenced channel *i* which was located between the *i*+1 and *i*−1 electrodes. The first and last contacts did not have neighbors, and were excluded from further analysis.

### Ripple Detection.

The LFP rereferenced signal was band-pass filtered between (80 to 250 Hz, zero-lag linear phase finite impulse response (FIR) filter), squared and z-scored normalized. The mean and SD was computed across the entire experimental duration to find the threshold for ripple event detection. Events from the normalized signal which exceeded 3.5 SD were selected as candidate ripples. Interripple interval periods of less than 5 ms were merged. In addition, ripples of less than 30-ms duration and more than 100 ms were excluded. Finally, candidate ripples which exceeded 5 SD were considered to be a ripple (12, 74, 75). To avoid false ripple detection, we added an additional constraint. After detecting ripples by the algorithm, we calculated the spectrogram of ripples using a Hanning taper window. There were some false detected ripples with peak frequency power of less than 80 or above 200 Hz. Those falsely detected ripples were excluded from further analysis.

CSD analysis: details are given in *SI Appendix*.

Response latency analysis: details are given in *SI Appendix*.

Laminar alignment: details are given in *SI Appendix*.

Cross-correlation of ripples between V1 and V4: details are given in *SI Appendix*.

### Number of Ripples in Behavioral Epochs and Peak Frequency of Ripples.

Ripples were detected in three periods in the attention task, namely i) precue, ii) postcue, and iii) sustained activity after stimulus onset. To quantify the ripple rate for a particular task epoch, the sum of ripples detected for a given epoch was divided by the product of trial number and the epoch duration for a given recording session [sum of ripples/(number of trials × epoch duration)]. The ripple rate was quantified separately for each session for all contacts combinations. Where relevant, statistics was performed over sessions, i.e., each session contributed a single ripple rate for a specific combination of conditions (e.g., precue; or e.g., during the sustained period: cue RF AND small stimulus AND narrow focus of attention; etc.). The peak frequency within a ripple was calculated in a 200-ms window centered on ripple events with a Hanning window. A KW test was used to determine whether peak frequencies differed between task epochs.

### Time–Frequency Spectral Modulation.

We estimated the power of the bipolar rereferenced LFP using the Chronux toolbox (76). Before Fourier transformation, the rereferenced LFP signal was meansubtracted and multiplied with a Hanning taper. We calculated the spectrogram of ripples using a Hanning taper window. Short Fourier transformation was performed on a 200-ms length sliding window over the data with a 5-ms overlapping step. The spectrogram was corrected by the baseline spectrum to remove the 1/*f* effect. We used wavelet-spectrograms to estimate comodulogram. The wavelet-spectrogram was computed using Fast Fourier Transform (FFT) of bipolar rereferenced LFPs convolved with a FFT of complex Morlet-wavelets. The output signal was acquired by the inverse FFT to estimate the time–frequency decomposition. The wavelet’s central frequencies ranged from 3 to 250 Hz in 40 logarithmically spaced steps (77).

### Spike Rate during Ripple Time.

Each recording channel raw data were filtered within the range of 600 to 9,000 Hz (sampled at 32,756 Hz). Spike sorting was done manually using SpikeSort3D (Neuralynx). We aimed to obtain a single unit for each contact, but given the nature of laminar recordings, this is often not possible across all contacts. We thus also included "small" clusters of multiunit activity. These sorted channels were used to generate stimulus-driven population histogram activity (*SI Appendix*, Fig. S14), which show activity levels similar to published data, where single electrode, single-unit activity has been reported (78). We then extracted and calculated unit activity for a given contact at ripple time within a 50-ms window centered at the ripple events across all attention conditions. To determine whether spiking activity varied with ripple events, we compared firing rates during ripples, with firing rates from the same number of trials where no ripples occurred, using matched time points within trials. For example, if a ripple occurred 412 ms after stimulus onset in one trial, we used the same time period in a condition/stimulus-matched trial where no ripple occurred. To ensure that results were robust to selecting different nonripple trials, we repeated the procedures 10 times. We found no systematic differences between these selections, and Fig. 5 shows activity averaged across the 10 repetitions (for no ripple trials).

### Statistical Testing.

#### Linear mixed-effects model.

We used Matlab’s fitlme function to perform a linear mixed-effect analysis of the relation between RTs or firing rate with task parameters including ripple occurrence (yes/no), attention to RF (cued RF/away), focus of attention (narrow/wide), and size of stimulus (large/small) across all trials of the monkeys. RTs of both monkeys were concatenated (i.e., pooled across sessions). Cueing location (RF/away), size of stimulus, focus of attention, and ripple occurrence were entered as fixed effects into the model as categorical variables, while different intercepts for each recording session were entered as random effects. Fixed-effect variables were defined as "categorical" in the model. RTs or firing rates were modeled as a linear combination of polynomial basis function of attentional cueing (*Att*), ripple (*R*) occurrence, focus of attention (*F*), and size of stimulus (*S*), where *β* is the polynomial coefficient.

$$y∼\beta_{0}+\beta_{1}Att+\beta_{2}R+\beta_{3}F+\beta_{4}S.$$

#### Ripple comparisons across task epochs.

KW tests were used to compare ripple duration and ripple peak frequency between task epochs. Wilcoxon sign rank tests were used to determine whether there was a significant difference between ripple rates on different attention conditions and attention blocks. A one-way ANOVA was applied to compare ripple rates between layers. An RM three-way ANOVA was performed on the interaction on task condition on ripple rate. For the comodulogram, we compared the power correlation using *t* tests. All multiple comparison were corrected using FDR (79), with an α < 0.05 as statistically significant, and *P*-values were adjusted as appropriate.

#### Ripples and cortical layers.

A one-way ANOVA (with different layer compartments as factors) was performed to determine differences in ripple rates between layers, with post hoc Tukey tests to determine pair-wise differences.

#### Effect of stimulus size on ripple events.

Wilcoxon signed rank tests were used to determine whether stimulus size affected ripple rates in V1 and/or in V4.

#### Relation of ripples to attention states, and possible interactions with stimulus properties.

Wilcoxon signed rank tests were used to determine whether cuing/attention (cue RF, cue away) conditions affected ripple rates in V1 and/or in V4 for the different stimulus periods, when stimulus size, or the focus size of attention was not factored out. To further explore whether ripple rate differed for attentional cue location (RF/away), the focus of attention (narrow/wide), and the stimulus size, a three-way RM ANOVA was used. Here, we determined whether a main effect for any of the three factors was present in a given area, and also whether significant interactions between the factors occurred. Where significant interactions (*P* < 0.05) did occur, we determined the "direction" of the interaction, i.e., which combination of factors differed from which other combination, by performing post hoc sign rank test (FDR corrected).

#### Spiking activity at ripple time.

To determine which factors of the task contribute to changed neural activity, we conducted a linear mixed-effect model analysis to predict the firing rate based on attention (RF/away), ripple occurrence (presence/absence), focus of attention (narrow/wide), and size of stimulus (large/small). We use the Matlab function fitlme.m to predict single trial firing rates based on factors listed above, followed by an ANOVA using the fitlme estimates.

#### Cross-correlation of V1 to V4 ripples.

Wilcoxon signed rank tests were used to determine whether the AUC relative to time zero showed significant lead-lag asymmetry.

#### Cross-frequency power coupling between ripples (comodulogram).

Power–power coupling, comodulogram, of ripples within and between V1 and V4 was computed using Pearson correlation coefficient between simultaneous wavelet-spectrograms for each brain area around the time of a ripple. We refer to the first signal as trigger and to the second signal as target. For instance, if a ripple was detected in a trial in V1, the spectrogram of the ripple (trigger spectrogram) and the spectrogram of the exact time in V4’s trial (target spectrogram) was computed. Afterward, in order to quantify the correlation of the two spectrograms, the Pearson correlation between the magnitude of power was calculated using the corr Matlab function (7, 80). The resulting correlation matrix was Fisher Z-transformed. This analysis was done across all contacts. The comodulogram was corrected by computing the comodulogram of random trials of the same conditions that did not contain ripples (matched for time during trial and exact condition combination). Finally, a *t-* test with FDR correction was conducted to find significant correlations (79). To estimate the comodulogram within an area, the same method was applied to calculate the power coupling between electrode contacts within and between layers.

#### Ripple rate and RT.

To determine whether RT were faster on trials where ripples occurred during the stimulus period, irrespective of any other experimental conditions, a two-sided *t* test was performed. To determine whether any RT differences were associated with ripples in V1, V4, or both, and possible interactions with additional experimental manipulations (location of cuing/attention, size of attentional focus, stimulus size) a linear mixed-effect model (fitlme.m, Matlab, followed by an ANOVA) was performed, yielding main and interaction effects between factors.

## Supplementary Material

Appendix 01 (PDF)

## Data Availability

Source data and analysis code are publicly available at (https://github.com/jafardm/Ripple_Attention_V1_V4). Data have been deposited to (https://gin.g-node.org/GeezleCode/Ripples_Attention_V1_V4) and published at G-Node (81).
