# Supplementary material for: Ripples in macaque V1 and V4 are modulated by top-down visual attention
Source: Proc Natl Acad Sci U S A. 2023 Jan 25;120(5):e2210698120. doi: 10.1073/pnas.2210698120 (PMC9945997; doi:10.1073/pnas.2210698120)
Supplement: Supplementary file 1 — Appendix 01 (PDF) [file pnas.2210698120.sapp.pdf]

PNAS
www.pnas.org

Supplementary Information for

Ripples in macaque V1 and V4 are modulated by top-down visual attention

Jafar Doostmohammadi[1,2,3], Marc Alwin Gieselmann[2], Jochem van Kempen[2], Reza Lashgari[4], Ali Yoonessi[1‡], and Alexander Thiele[2*]

[1] Department of Neuroscience and Addiction studies, School of Advanced Technologies in Medicine, Tehran University of Medical Sciences, Tehran, Iran.

[2] Biosciences Institute, Newcastle University, NE1 7RU, Newcastle upon Tyne, United Kingdom

[3] School of cognitive sciences, Institute for Research in Fundamental Sciences, IPM, Tehran, Iran

[4] Institute of Medical Science and Technology, Shahid Beheshti University, Tehran, Iran

*Corresponding authors: Alexander Thiele, Biosciences Institute, Newcastle University, Newcastle upon Tyne, NE2 4HH.

Email: alex.thiele@ncl.ac.uk ORCID ID: https://orcid.org/0000-0003-4894-0213

‡ Co-corresponding authors: Ali Yoonessi

This PDF file includes:

Supplementary text
Figures S1 to S15
Tables S1 to S9
SI References

Supplementary Information Text

Supplementary Methods

Visual stimulation:
Visual stimuli were generated on a personal computer and presented on a CRT monitor (Monkey 2: Dell Multiscan P1110, 21'', mean luminance=90cd/m2; Monkey 1: Iiyama HM204DTA, 22'', mean luminance=53cd/m2) with a refresh rate of 120 Hz, and a resolution of 1024x768 pixels. At the beginning of each session the monkeys' eye position was calibrated. The eye position was monitored using an optical eye-tracker (ET-49, Thomas Recording, Giessen, Germany) with a sampling frequency of 200 Hz. Stimulus presentation and behavioral control was handled by Remote Cortex 5.95 (Laboratory of Neuropsychology, National Institute for Mental Health, Bethesda, MD).

Data acquisition
Electrophysiological recordings were performed using passive laminar probes with 16 recording contacts, inter-contact spacing of 150 µm (ATLAS Neuroengineering, Belgium). The laminar probes were inserted perpendicularly to the cortical surface with the support of a hydraulic micromanipulator (NARISHIGE MO-97A, Japan). Data were recorded using a digital acquisition and control system (Digital Lynx, Neuralynx, USA) with a sampling frequency of 32556 Hz (~32 kHz), at 24 bits.

Basic Response Characterization (Receptive Field Mapping)
Prior to starting the attention paradigm, the classical receptive field was determined using a reverse-correlation paradigm, as described in detail previously (11). Briefly, dark squares with a size of 1º, 0.5º or 0.25º were used for mapping of receptive fields. Michelson contrast of the stimuli was 95-98 %. Stimuli were presented for 108 ms (monkey 1) or 125 ms (monkey 2) and covered a 9 × 12 grid of multiples the respective stimulus size.
RF maps were initially estimated online based on thresholded MUA spiking activity to determine the stimulus locations in the attention paradigm. To acquire the envelope of multiunit activity (MUA_E), a Butterworth (4rd order, 0.6-9 kHz) was applied on the raw signals and rectified. The envelope of the resulting signal was computed by a low-pass filtering (Butterworth 5rd order, <300 Hz) and down sampled to a frequency of 1017 Hz (12). Offline RF analysis was done based on local MUA_E, using a time window from 40-120 ms after RF mapping stimulus onset.
For each mapping stimulus we averaged MUAe activity in a window of 40-120 ms post stimulus onset and computed the z-score, relative to a window before stimulus onset (-50-0 ms). We used the z-score of each stimulus position (12 by 9 grid) to generate RF maps for each of the 16 probe contacts. The RF maps were then upscaled by a factor of 10 using bicubic interpolation. For each map the position with the maximum z-score (>1) was defined as the RF-center. The RF-area was then defined as a 36-point polygon around all positions around the RF-center with a z-score of at least 1. The RF-size is given as square degrees visual angle.

We also determined orientation selectivity prior to the main recording sessions, and stimuli used were matched for the aggregate orientation preference obtained in a session where possible.

Response Latency analysis
To estimate the latency of visual responses we used a method described previously (1). It makes two assumptions; (i) namely that the onset of the neuronal response has a Gaussian distribution across trials and (ii) that a fraction of response modulations dissipates exponentially after reaching the peak magnitude. These assumptions yield a function *f(t)* which consists of an ex-gaussian and a cumulative Gaussian function.

$$f(t) = d.e^{\mu\alpha+\left(\frac{\sigma^2\alpha^2}{2}\right)-\alpha t}.G(t,\mu+\sigma^2\alpha,\sigma)+c.G(t,\mu,\sigma)$$

The parameters of μ and σ are the mean and standard deviation of the cumulative Gaussian, which are determined by onset response time. The parameter α is the dissipating rate and *c* and *d* act as weighting factors for the response magnitude and dissipation terms.

For our data, we acquired 150 ms of MUA_E signal after stimulus onset, which was z-scored to baseline activity (before stimulus onset). The function *f(t)* was fit to the z-scored activity. The latency of visual responses (lat_{33}) was determined as a point in time where the fitted function reached 33% of its maximum (1, 2).

Current source density (CSD) analysis

We used the inverse CSD (iCSD) toolbox to compute CSDs. CSD analysis was calculated by applying the spline method (3). LFPs were averaged over trial repetitions. Then, the iCSD, which is the second spatial derivative voltage (Φ) of LFPs, can be approximated using the following formula:

$$CSD(z) = -\sigma\,\frac{\Phi(z+h)-2\Phi(z)+\Phi\,(z-h)}{h^2}$$

Where *z* is the depth at which the CSD is estimated, and *h* is the space between two electrodes (150µm). With this toolbox we used a Spline fitting method to interpolate Φ smoothly between electrode contacts. In our computation we assumed a tissue conductivity (σ) and a cortical column radius of 0.4 s/m and 500µm respectively (4, 5).

Laminar alignment

The CSD was used for alignment of probe contacts to the cortical layers with reference to layer IVc. Previous studies (6–8) established that an early sink in the CSD profile corresponds to input layers, with associated current inflow. First, we computed the CSD across all stimulus presentations of each session (Supplementary figure S7A) and visually determined the contact that featured the early sink (35-55 ms after stimulus onset; Supplementary figure S7B). Additionally, we determined the channel/contact with the shortest latency stimulus-evoked response MUA_E response (details in supplementary materials and supplementary figure S7C, D). Using these criteria, we assigned the reference contact as input layers (presumably layer IVc in V1, and layer IV in V4) and signals from other contacts were assigned to superficial, input and deep layers depending on their distance from the reference contact. For area V1, channels at 0.25 mm above and 0.25 mm below the reference channel were labelled as input layer (presumably layer IV), channels at 0.25 mm to 1 mm above the reference were labeled as superficial layer (presumably I, II, III) and channels below the reference channel at 0.25 mm to 0.75 mm were labeled as deep layer (presumably V, VI) (9, 10). For V4, contacts less than 0.1 mm above and below the reference contact were identified as input, 0.1 to 1 mm above the reference contact were identified as superficial, and those 0.1 mm to 0.75 mm below the reference channels were labelled as deep layers (Supplementary figure S7, C, D). Channels outside these ranges were excluded from further analyses.

Cross-correlation

The temporal relationship between ripples that occurred in V1 and V4 was explored using cross-correlation analysis. The cross-correlation (CC) was performed using the xcorr function in Matlab, according to:

$$CC = \frac{1}{M}\sum_{1}^{M}\frac{\sum_1^T x(t)y(t+\tau)}{\sqrt{\sum_1^T |x(t)|^2 . \sum_1^T |y(t)|^2}}$$

Where x and y are vectors representing ripple occurrence in area V1 and V4. To compute the cross-correlation, the envelope of the LFP was extracted. For each trial, the LFP of the sustained period was filtered at ripple band (100-200 Hz, Butterworth, 4th order) and rectified to convert negative values to positive. Using the Hilbert transform, the envelope of the signal was calculated and smoothed by a 4th order Butterworth filter (1-20 Hz). The cross-correlation coefficient of V1 and V4 trials was computed and corrected by the shuffling of V4's trials. M and T denotes the number of trials and discrete time bins respectively and t represents the time lag. The area under the cross-correlation curve above the shuffle predictor was calculated for the left and the right part of the cross correlogram relative to time zero (AUC), and it was used to examine whether ripple occurrence between V1 and V4 was on average simultaneous, or whether it was systematically shifted in time for one of the two areas.

Ripple rate and RF overlap

To assess whether V1 and V4 RF overlap, and therefore stimulus placement, affected the ripple rate, we classified recording sessions into two categories. Namely, where V1 RFs were completely or almost completely covered by all of the V4 RFs (these are labelled as 'fully overlapped', examples in figure S9) and sessions where the overlap was less complete ('partial overlap', example in figure S9). Then, we quantified ripple rate for sessions with full and partial RF overlap. A one-way ANOVA revealed that there was no difference in ripple rate between sessions with full and partial overlap. This was the case for both V1 and V4 ($F(1,732) = 1.57$, $p = 0.21$, pooled data and figure S10 panel A). Furthermore, we explored whether ripple rate modulation by stimulus size would change depending on RF overlap between V1 and V4. The reasoning here is that if small stimuli are centered on V1 RFs, which do not fully overlap with V4 RFs, V4 RFs would not, or only partially be driven by the small stimulus. This would not be the case if V1 and V4 have full overlap.

We explored the ripple rate among the fully overlapped sessions (n=18) in different task conditions. Corroborating the results reported in the main manuscript (where data were pooled across all session), small stimuli elicited a higher ripple than large stimuli in both areas. For these 18 recordings ripple rate for small and large stimuli were 0.15 ES and 0.09 ES in V1. In V4, rates were 0.11 ES and 0.05 ES for small and large stimuli (V1: $Z = -4.2$, $p < 0.001$. V4: $Z = -7.01$, $p < 0.001$, Wilcoxon's signed rank test, figure S10 panel B). In addition, attention to RF enhanced ripple rate when compared to cue away conditions among these sessions. Cue RF and away conditions in V1 triggered 0.13 ES and 0.10 ES respectively. In V4 cue RF conditions triggered ripple rates of 0.12 ES while cue away conditions elicited 0.05 ES (V1: $Z = 2.08$, $p = 0.03$. V4: $Z = 8.11$, $p < 0.001$, figure S10 panel C). We conducted a 3-way repeated measures ANOVA, to determine main effects of stimuli, attentional location and attentional focus, and possible interactions on ripple rate. ANOVA revealed that stimulus size ($p=0.005$) and attention location ($p=0.02$) had main effect on ripple rate frequency in V1 data. There was a significant attention location*size interaction ($p=0.04$) and attention location*attentional focus interaction ($p=0.02$) in V1. The 3-way ANOVA in V4 showed that stimulus size ($p=0.0009$) and attention location ($p<0.001$) significantly increased ripple rate. We found a stimulus size and attention location interaction on ripple rate ($p=0.0003$) in V4.

Data Analysis software

- MATLAB® 2018b (Mathworks Inc, USA).
- Neuralynx MATLAB-Netcom Utilities 6.0.0 (Neuralynx Inc., USA).
- Freely Moving Animal toolbox (http://fmatoolbox.sourceforge.net).

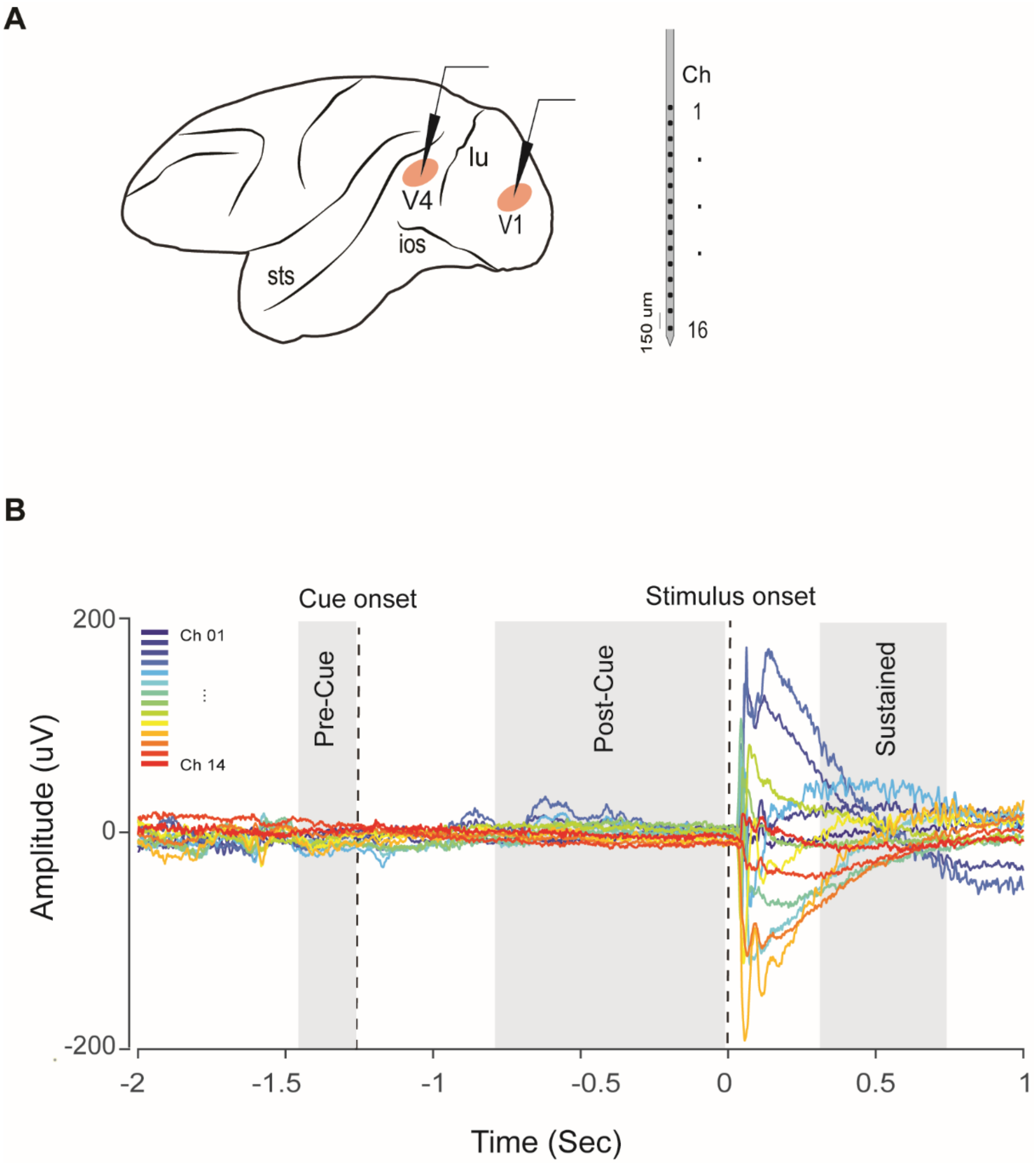

Fig. S1. Laminar probe and event related potential (ERP). A) Schematic representation of 16-channel laminar probe inserted into V1 and V4 for collecting of spikes and LFPs. B) Traces of ERPs acquired by the probe and task epochs used for detection of ripples. Each color depicts average of a bipolar re-referenced LFP acquired by a contact. Time of epochs aligned to stimulus onset (time 0). Black dashed lines denote the onset and offset of cue and stimulus. Numbers indicates contact's number across depth of cortex, with upper layers being at the top.

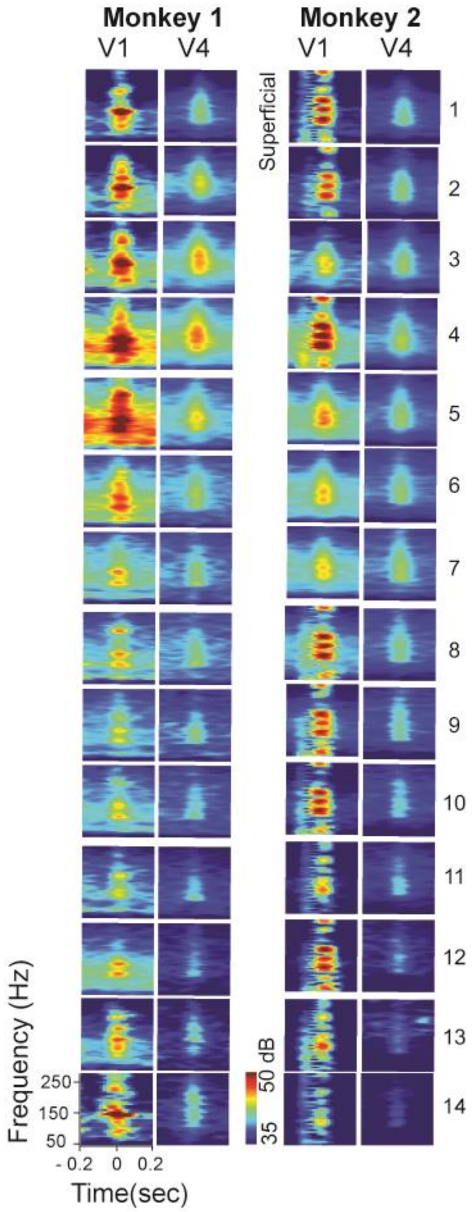

Fig. S2 Depth profile of spectrogram of ripples across electrode contacts. Left panel indicates grand average of spectrogram of ripples identified on bipolar-referenced LFPs acquired by laminar probes in Monkey 1 in V1 and V4. Right panel shows data for monkey 2. Numbers indicates contact's number across depth of cortex, with upper layers at the top.

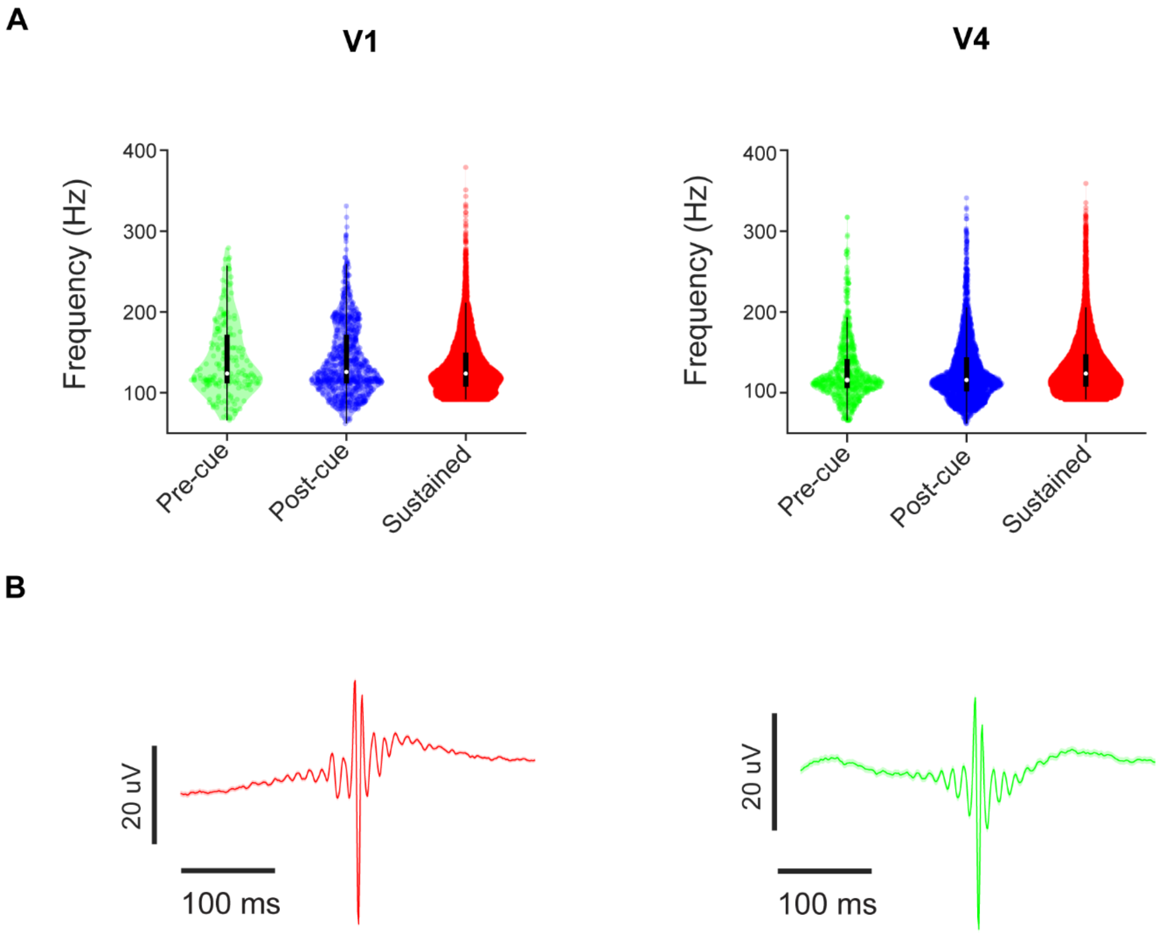

Fig. S3. Ripple's peak frequency and mean peri-ripple field potentials in V1 and V4. A) Peak frequency of ripples among pre-cue, post-cue and sustained intervals. Ripples peak frequency in V1 and V4 was calculated from a 200 ms window centered at ripple time. Ripples in the sustained period showed lower peak frequency compared to pre-cue and post-cue conditions in V1 as well as V4. B) Group-averaged ripple-triggered wide-band traces (<300 Hz) for V1 and V4. The white circle indicates median and black rectangle representing first and third quartile of the data.

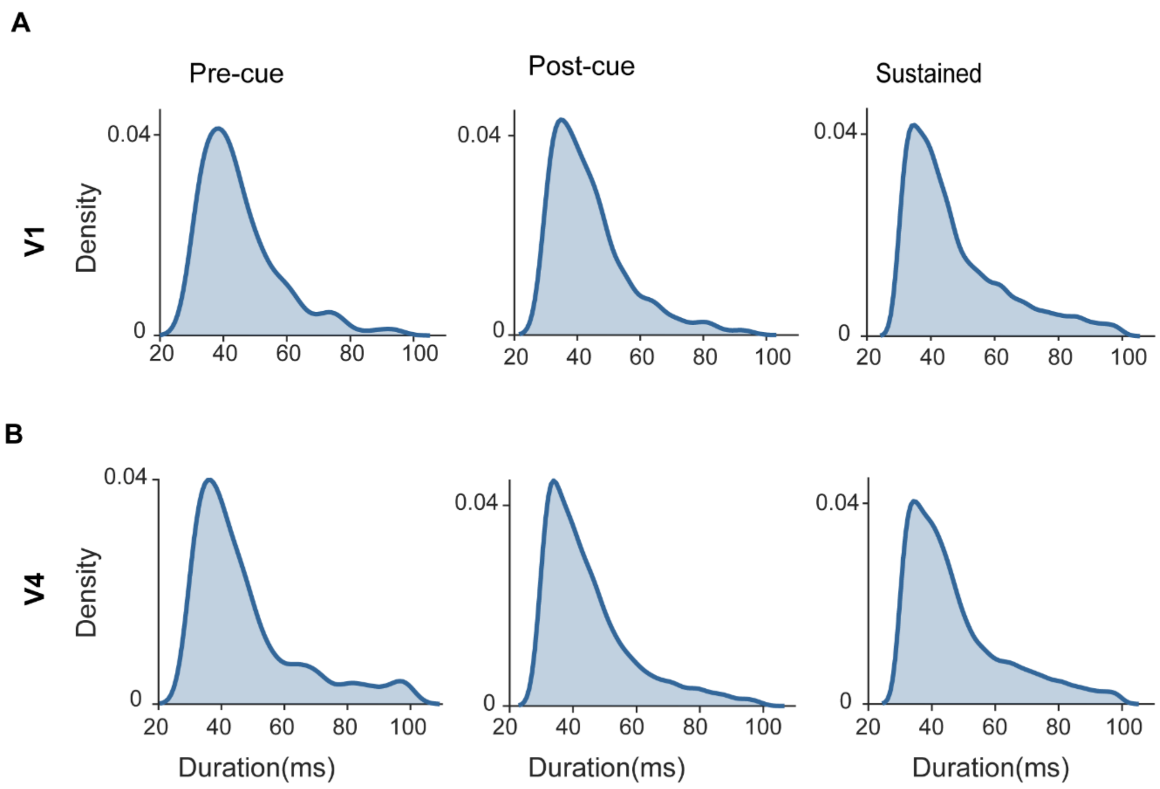

Fig. S4. Kernel density of ripple duration during different task epochs for V1 and V4. Ripple duration was longer during the sustained period than post and peri cue periods in V1 and V4.

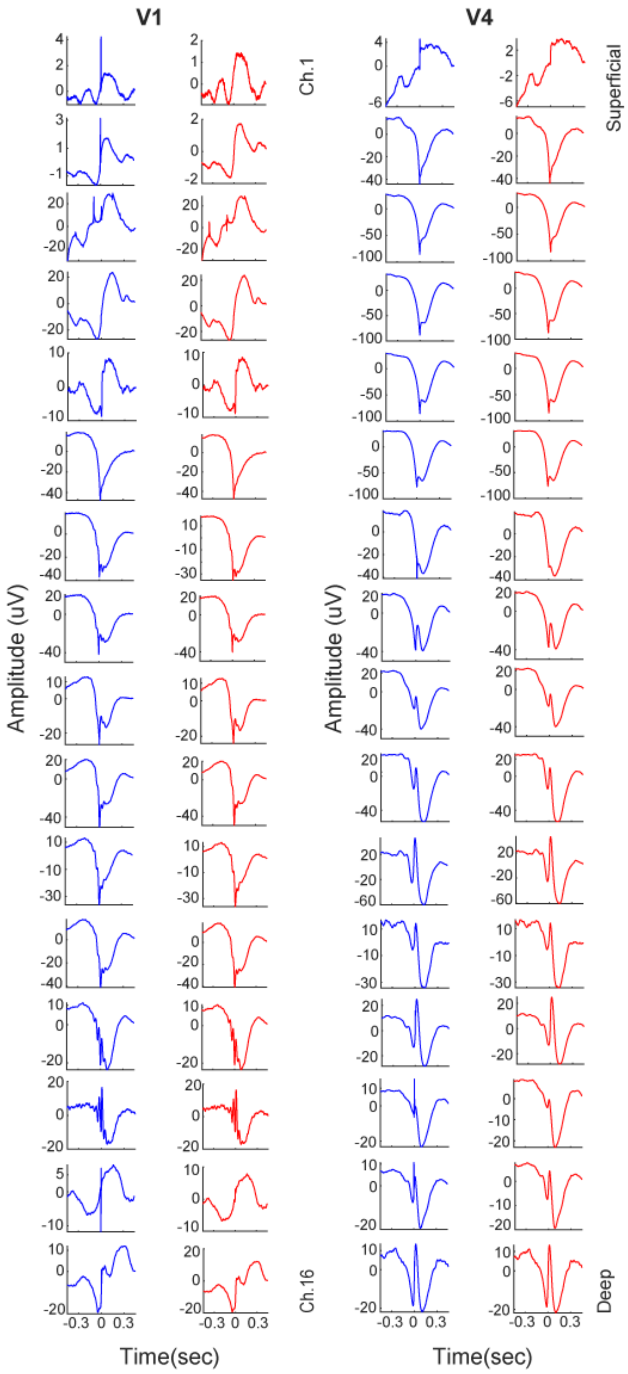

Fig. S5. Spike triggered average LFP of a sample data (recording across 16 contacts) of V1 (left column) and V4 (right column). Left column shows LFP (< 300 Hz) recorded from a 16-channel electrode from V1. Blue color represents STA of LFP signal for each channel without de-spike procedure. Red color indicates the same channel after removal of spike leakage by Bayesian estimate method. Right column shows data for V4. Same as V1, blue and red are STA before and after de-spiking. Order of channels is across depth of cortex, with upper layers at the top.

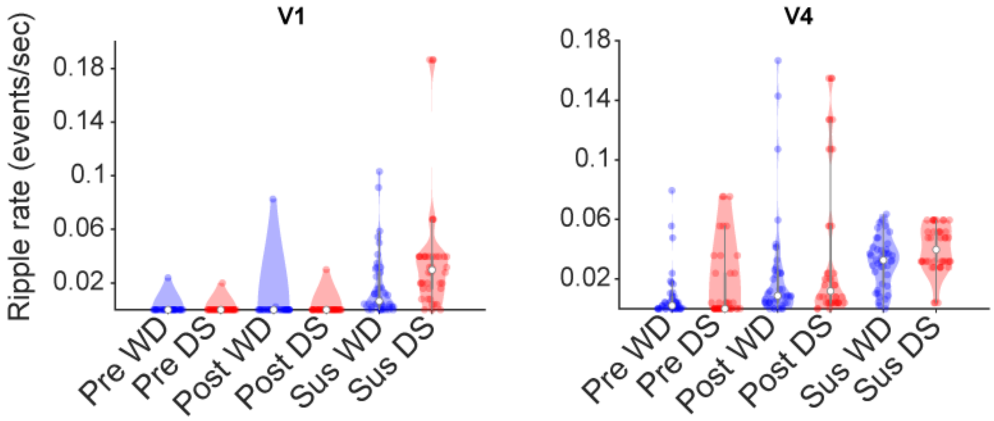

Fig.S.6. Comparison of ripple occurrence rate on the LFP data before and after de-spiking. Left panel shows ripple rate in V1 computed on LFP data without de-spiking (blue color), and ripple detected on the LFP which potential spike leakage had removed by Bayesian estimate (red color) for pre-cue, post-cue and sustained periods. Right panel shows ripple rate for different task conditions in V4. Like V1 blue and red color represent ripple rate without and with de-spiking LFP. Pre post and sus corresponds to pre-cue, post-cue and sustained condition. WD and DS stands for without de-spike and de-spiking data. Wilcoxon signed rank test revealed sustained period on the de-spike data expressed higher ripple rate than the method used here (V1: $z = -4.11$, $p < 0.001$; V4: $z = -2.5$, $p = 0.01$).

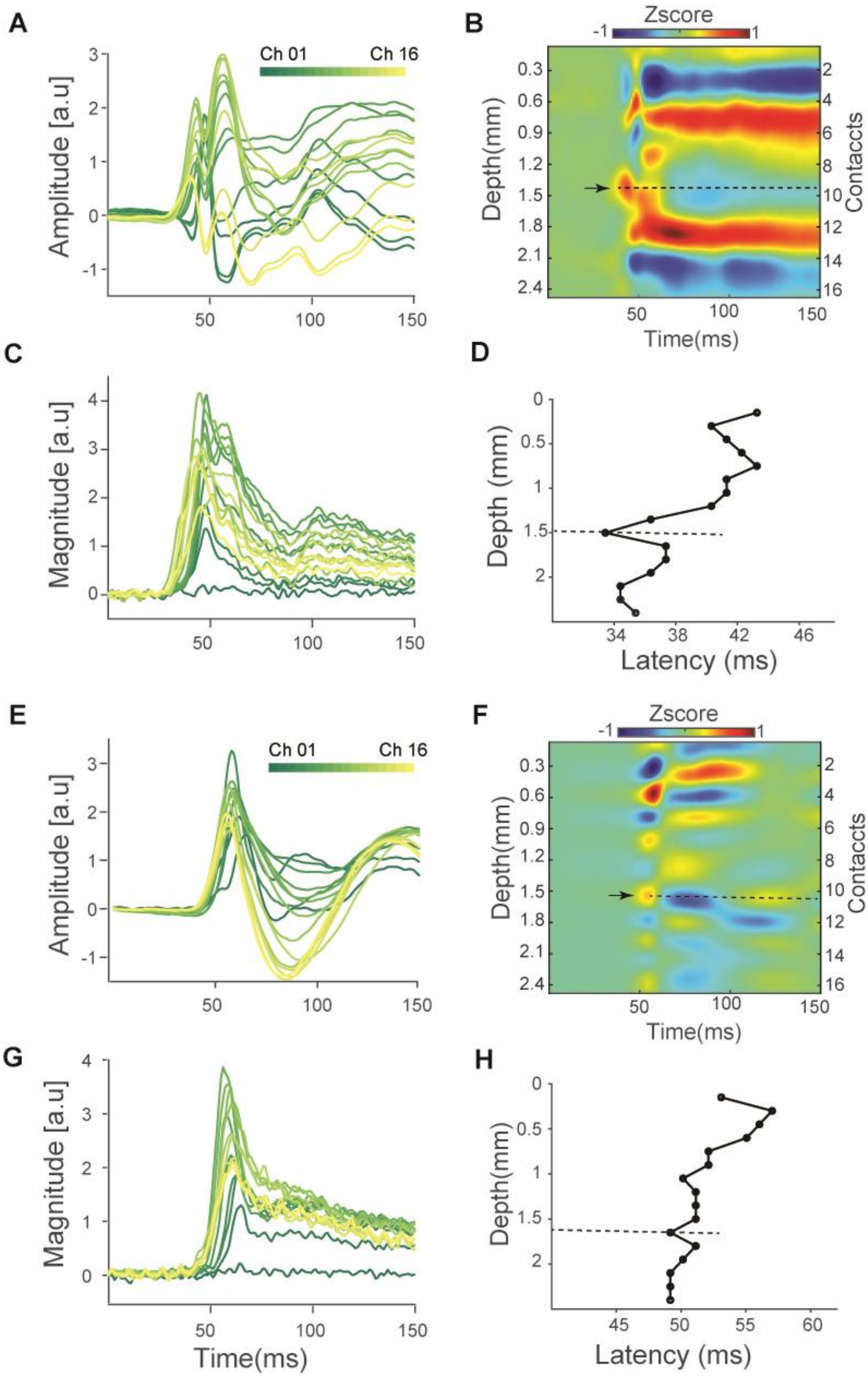

Fig. S7. Samples of laminar alignment in V1 (A to D) and V4 (E to H). A, E) LFPs z-scored to the baseline activity. Different colors denote activity acquired by different contacts. C, G) MUA_E z-scored to baseline activity used to compute latency of each contact. B, F) CSD z-scored to baseline. Black arrow represents the first input to the cortex (sink region). The dotted line is the depth defined for alignment of

the layers. D, H) are response latency indices across depth. Channel contact's number are across depth of cortex, with channel 01 and upper layers at the top.

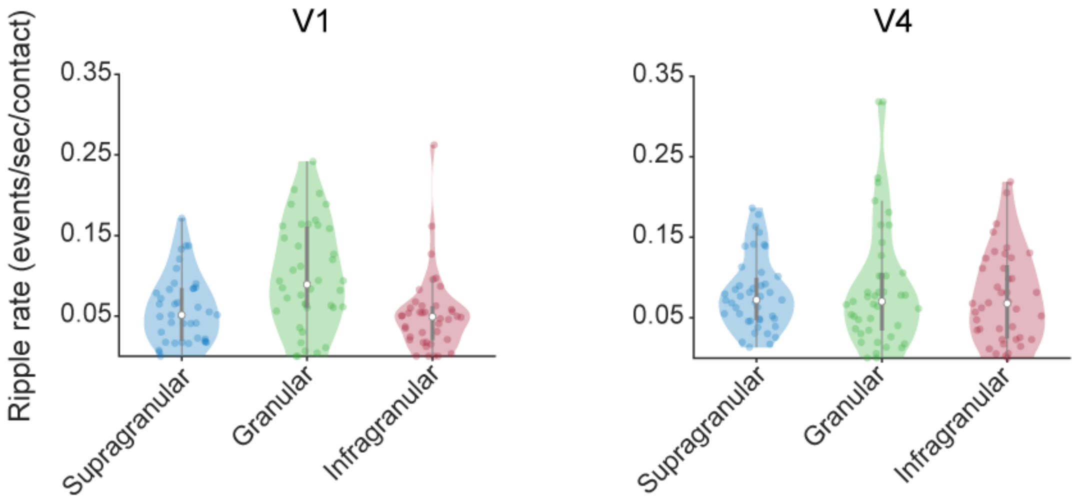

Fig. S8. Ripple rate across cortical depth. Left panel illustrates ripple rate for V1, and right panel represents ripple rate for V4 layers (per contact) for superficial, input and deep layers. Ripple rate was larger for input layers in V1 compared with deep layers but did not differ otherwise between compartment comparisons. The white circle indicates median and grey rectangle representing first and third quartile of the data.

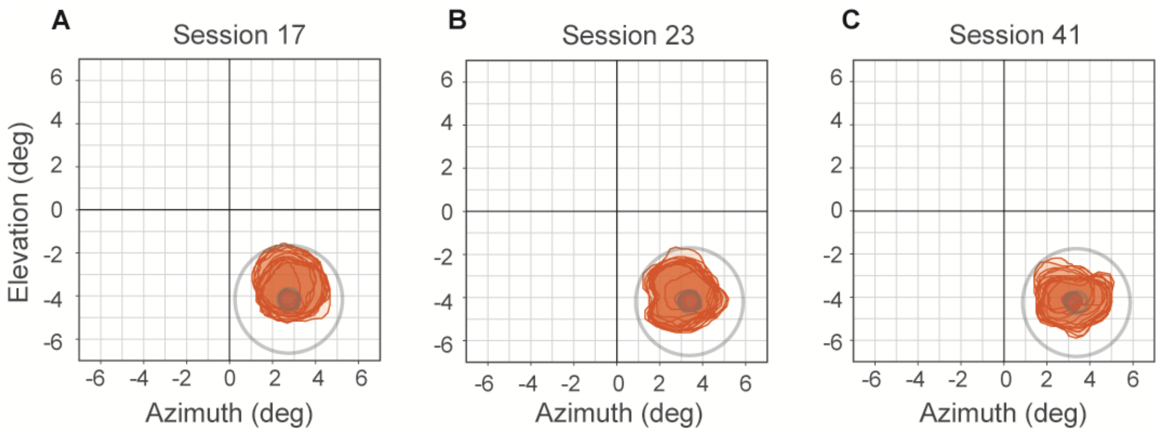

Fig. S9. Illustration of three sample recordings with fully overlapping V1 and V4 receptive fields. Panels depict V1 and V4 RFs obtained from MUA_E activity for each electrode contact using 0.25º, 0.5º and 1º sized stimuli. RF contours are relative to fixation point (point 0,0, units are in degrees of visual angle). Blue colors are V1 RFs, covered by V4 RFs in red.

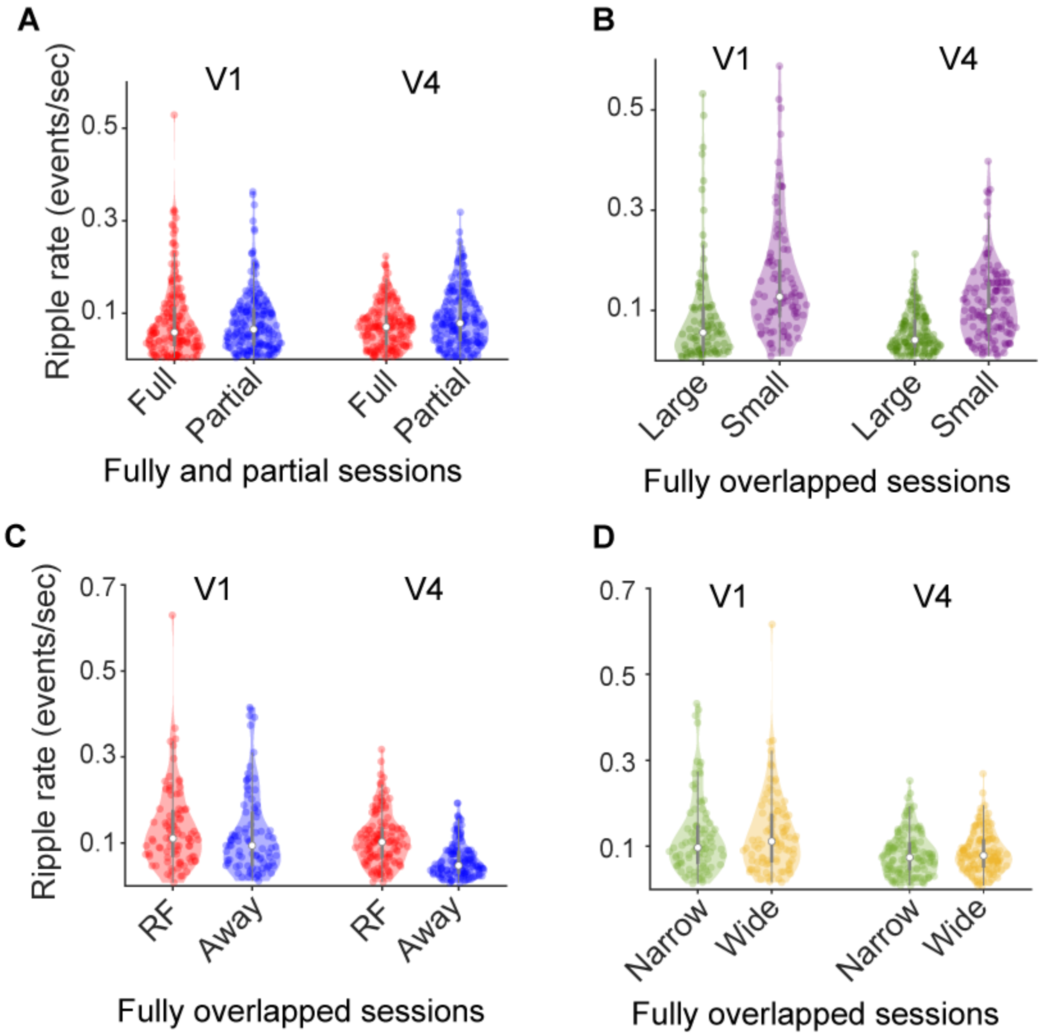

Fig. S10. Ripple rate with respect to receptive field (RF) overlap between V1 and V4. A) Comparison of ripple rate as function of overlapping of V1 and V4 RFs. Red and blue colors represent ripple rate quantified between recordings where RFs of V1 were completely (full) or partially (partial) overlapping with V4 RFs. B) Effect of stimulus size on ripple rates for recordings with full overlapping V1 and V4 RFs. C) Ripple rate in attend to RF/away conditions for session with full RF overlap. Similar to the results reported in the main manuscript, sessions with full V1-V4 RF overlap, show that small stimuli and cue RF elicited higher ripple than large and cue away condition. This effect was consistent in V1 and V4. D) Ripple rate computed on the narrow and wide blocks among the sessions with fully overlapping of the RFs in V1 and V4. The white circle indicates median and grey rectangle representing first and third quartile of the data.

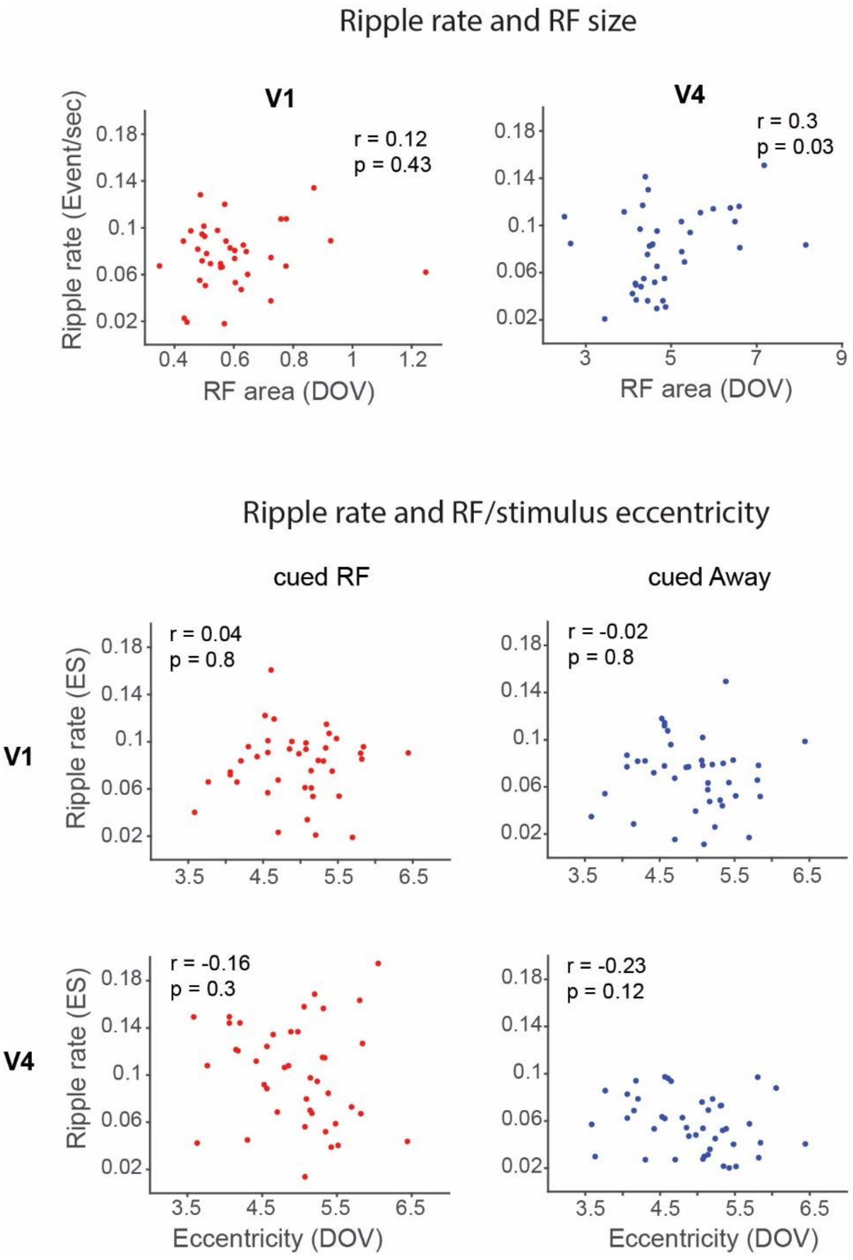

Fig. S11. Ripple rate with respect to receptive field (RF) size and eccentricity. Top) Ripple rate plotted against RF size for V1 and V4. RF size was calculated as described under 'receptive field mapping' above. Middle and bottom) Ripple rate plotted against RF eccentricity for V1 and V4 and cued RF and cued away conditions. RF eccentricity was calculated as the vector length distance between the fixation spot and the centre of the RF (described in 'receptive field mapping'). R-values (r) and associated significances (p) indicate Pearson correlations between the respective variables. Only for V4 a small systematic relationship between RF area and ripple rate was apparent.

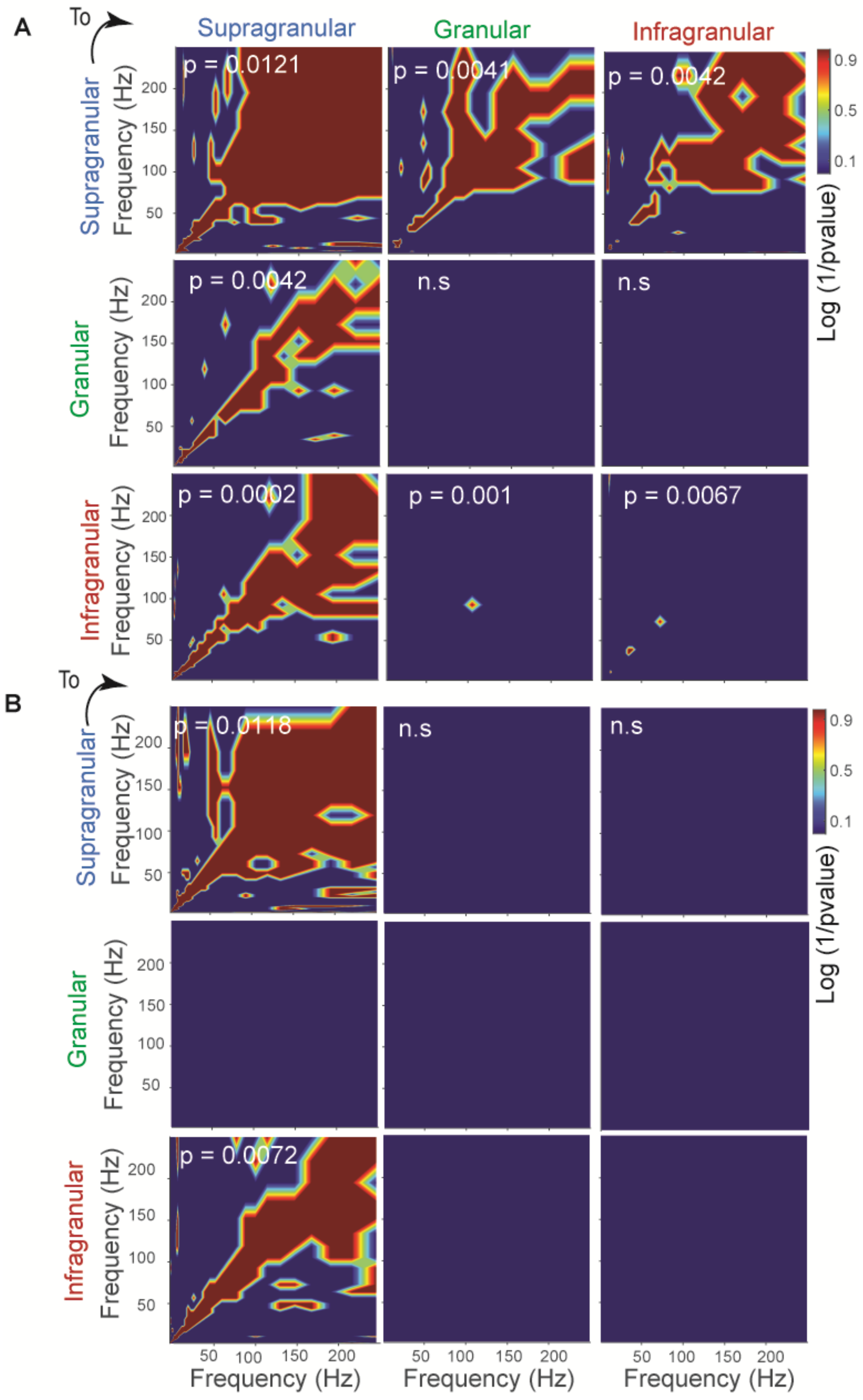

Fig. S12. Within layer power correlation. A) Corrected comodulogram between superficial, input and deep layers in V1. Each panel shows distribution of p-values, log10(1/p-values), between comodulogram of layers where a specific layer served as a trigger. The arrow at top left denotes the direction of the trigger

region and the respective response. Red areas show frequency bands that showed significant power coupling (t-test with FDR correction). B) Comodulogram of ripple in V4 between different layers. Same as (A) but for area V4.

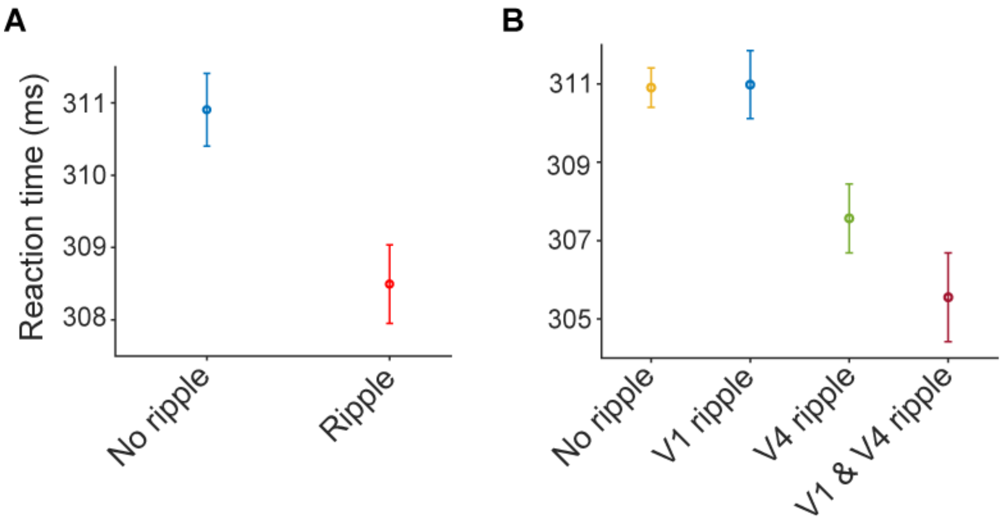

Fig. S13. Reaction time across trials with and without ripples in V1 and V4. A) Red and blue indicate mean and SEM of RTs of trials with and without ripples regardless of task conditions accumulated for both subjects. B) Mean and SEM of reaction time during trials when no ripples occurred during the sustained period, when ripples occurred only in V1 during the sustained period, when they occurred only in V4 and when the occurred in both areas, V1 and V4.

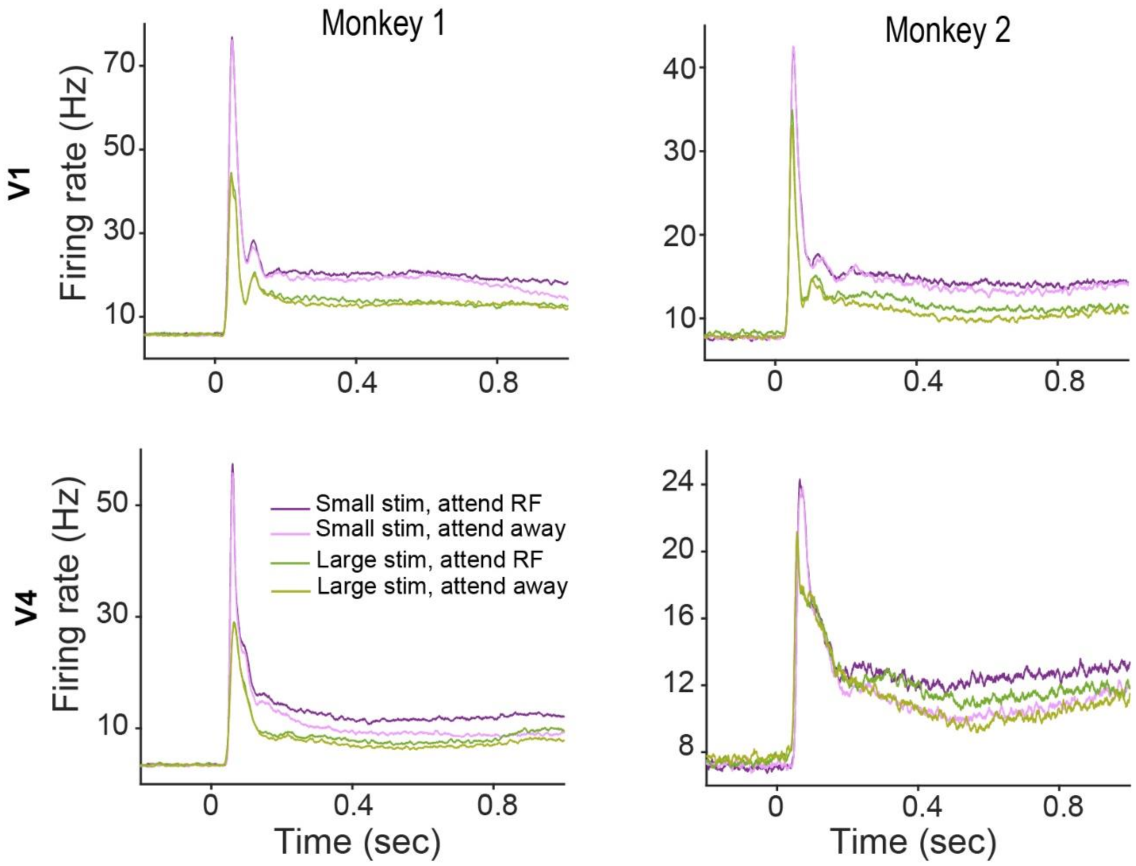

Fig S.14. **Firing rates associated with small (purple/pink) and large stimuli (green), as well as cue RF and cue away conditions in V1 and V4.** In both areas, small stimuli elicited higher firing rates than large stimuli. In addition, cue RF conditions elicited higher firing rates than cue away conditions during the sustained stimulus period.

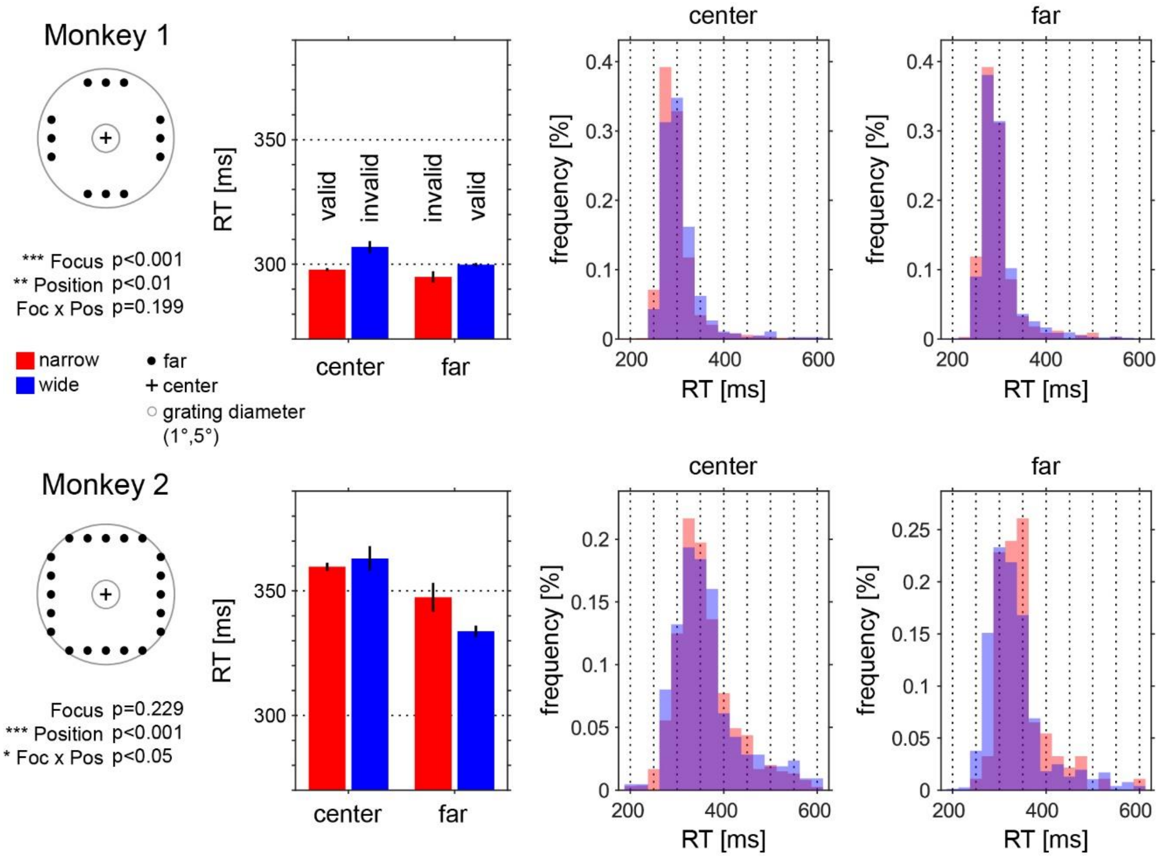

Fig. S15. **Reaction times associated with wide and narrow foci of attention and with valid and invalid cueing.** During narrow blocks of attention (red bars and histograms) targets were presented in ~10% or ~7% (M1 or M2) of the trials at far positions (invalid; possible target locations indicated by black dots inside the grey circles which represent small and large stimuli respectively). Conversely during wide blocks of attention targets were presented in ~10% or ~7% (M1 or M2) of the trials at the centre position (invalid). Thus, we would expect for a given target location that validly cued targets yield shorter reaction times than invalidly cued targets. While this was the case in both monkeys if the targets appeared at the centre, we only found that pattern for far targets in monkey 1. In monkey 1 narrow foci of attention always yielded faster reaction times for a given target location, compared to wide foci of attention. Thus, we found a significant main effect of focus and position, but no interaction on reaction times in that monkey (2- factor ANOVA). However, in monkey 2 we found a significant interaction between target position and focus of attention, in line with both predictions.

	Monkey 1			Monkey 2			Monkey 1&2		
	**V1**	**V4**	**V1 & V4**	**V1**	**V4**	**V1 & V4**	**V1**	**V4**	**V1 & V4**
Session	25	26	25	15	17	13	40	43	28
Correct trials	10136	10762	10136	7897	9058	7113	18033	19820	17249

Table S1. Recording sessions and trials.

Number of sessions for data recorded in V1 and in V4. The columns V1&V4 report the number of sessions and trials where data were recorded simultaneously in both V1 and V4.

Table S2: Three-Factor (Stimulus, Location of Attention, Attentional Focus) Analysis of Variance with Repeated Measures to assess modulation of V1 ripple rate (during the sustained period)

	SS	df	MS	F	P
Between-Sessions	0.419	39			
Within-Sessions	1.594	280			
**Stimulus**	0.560	1	0.560	31.233	<0.001
error (stimulus)	0.530	39	0.014		
**Attention**	0.016	1	0.016	6.564	0.014
error (attention)	0.096	39	0.002		
**Focus**	0.000	1	0.000	0.016	0.902
error(focus)	0.075	39	0.002		
**Stimulus* Attention**	0.006	1	0.006	4.796	0.035
Error(stimulus*attention)	0.176	39	0.004		
**Stimulus*Focus**	0.036	1	0.036	25.765	<0.001
Error(stimulus*focus)	0.040	39	0.001		
**Attention*Focus**	0.012	1	0.012	7.562	0.009
Error(attention*focus)	0.043	39	0.001		
**Stimulus*Attention*Focus**	0.006	1	0.006	0.150	0.7476
Error	0.055	39	0.001		
**Total**	1.978	639			

3-way repeated measures ANOVA to determine whether ripple rate in area V1 during the sustained period depended on stimulus size (small/large), locus of attention (cue RF/away) and attentional focus size (narrow/wide). Table denotes sum of squares (SS), degrees of freedom (df), mean squares (MS), F-value (F), and p-value (P). Interaction terms are indicated by * sign.

Table S3: Three-Factor (Stimulus, Location of Attention, Attentional Focus) Analysis of Variance with Repeated Measures to assess modulation of V4 ripple rate (during the sustained period)

	SS	df	MS	F	P
Between-Sessions	0.711	42			
Within-Sessions	2.288	301			
**Stimulus**	0.530	1	0.530	31.233	<0.001
error (stimulus)	0.712	42	0.017		
**Attention**	0.378	1	0.378	110.428	<0.001
error (attention)	0.144	42	0.003		
**Focus**	0.011	1	0.011	8.681	0.005
error(focus)	0.052	42	0.001		
**Stimulus* Attention**	0.142	1	0.142	33.830	<0.001
Error(stimulus*attention)	0.176	42	0.004		
**Stimulus*Focus**	0.008	1	0.008	7.871	0.008
Error(stimulus*focus)	0.040	42	0.001		
**Attention*Focus**	0.007	1	0.007	6.810	0.013
Error(attention*focus)	0.043	42	0.001		
**Stimulus*Attention*Focus**	0.002	1	0.000	0.048	0.8282
Error	0.036	42	0.001		
**Total**	2.991	687			

3-way repeated measures ANOVA to determine whether ripple rate in area V4 during the sustained period depended on stimulus size (small/large), locus of attention (cue RF/away) and attentional focus size (narrow/wide). Table denotes sum of squares (SS), degrees of freedom (df), mean squares (MS), F-value (F), and p-value (P). Interaction terms are indicated by * sign.

Table S4. Multiple linear mixed effect model results to predict firing rates in V1 from attention, ripples, attentional focus and stimulus size.

Predictors (V1)	β	T	p-values
Ripple (0)	- 4.1	- 5.3	< 0.001***
Attention (1)	- 0.5	- 0.7	0.4
Focus (1)	-1	-1.2	0.1
Size (1)	0.6	0.8	0.4
Size (2)	7.2	9.2	< 0.001***
Ripple (0) * attention (1)	0.2	0.2	0.7
Ripple (0) * Focus (1)	0.7	0.9	0.3
Attention (1) * Focus (1)	0.7	0.9	0.3
Ripple (0) * size (1)	0.9	1.1	0.2
Ripple (0) * size (2)	-0.0005	-0.0007	0.9
Attention (1) * size (1)	0.5	0.7	0.4
Attention (1) * size (2)	0.6	0.8	0.4
Focus (1) * size (1)	0.3	0.4	0.6
Focus (1) * size (2)	0.5	0.6	0.5
Ripple (0) * attention (1) * Focus (1)	-0.4	-0.5	0.5
Ripple (0) * attention (1) * size (1)	0.02	0.03	0.9
Ripple (0) * attention (1) * size (2)	-0.2	-0.3	0.7
Ripple (0) * Focus (1) * size (1)	-0.9	-1.1	0.2
Ripple (0) * Focus (1) * size (2)	-0.4	-0.6	0.5
Attention (1) * Focus (1) * size (1)	-0.4	-0.6	0.5
Attention (1) * Focus (1) * size (2)	-0.9	-1.1	0.2
Ripple (0) * Attention (1) * Focus (1) * size (1)	0.4	0.5	0.5
Ripple (0) * Attention (1) * Focus (1) * size (2)	0.6	0.7	0.4

Linear regression model to predict V1 firing rate from occurrence of ripple, attention to RF vs away, size of focus of attention (narrow/wide), and size of stimulus (small/large). The digits in front the predictors represent the categories assigned to fixed effects into the model. Ripple (0 is without and 1 with ripple), attention (1 attention to RF 2 and away), focus (1 narrow and 2 wide focus of attention) and size (1 large and 2 small stimuli). The column of β reports the coefficients of each predictor estimated by model and column T is the corresponding t-statistic. Asterisks indicate significance.

Table S5. Mean and SEM of firing rate across trials with and without ripple and during different attention conditions in V1.

Ripple	Attention	Mean ± SEM of firing rate
No	RF	21.8 ± 0.2
No	away	21.5± 0.2
Yes	RF	29.6 ± 0.3
Yes	Away	29.3 ± 0.3

Column 1 indicates ripple occurrence in trials (with: yes; without: no ripple). Column 2 attention indicates attend to RF/away from RF condition.

Table S6. Multiple linear mixed effect model in V4.

Predictors (V1)	β	T	p-values
Ripple (0)	-4.3	-3.3	< 0.001***
Attention (1)	0.4	0.3	0.7
Focus (1)	0.8	0.6	0.5
Size (1)	1.1	0.8	0.4
Size (2)	3.2	2.4	0.01 **
Ripple (0) * attention (1)	0.2	0.1	0.8
Ripple (0) * Focus (1)	0.6	0.4	0.6
Attention (1) * Focus (1)	-0.3	-0.2	0.7
Ripple (0) * size (1)	-0.6	-0.4	0.6
Ripple (0) * size (2)	0.06	0.05	0.9
Attention (1) * size (1)	-0.4	-0.3	0.7
Attention (1) * size (2)	0.7	0.5	0.5
Focus (1) * size (1)	-0.2	-0.2	0.8
Focus (1) * size (2)	-1.2	-0.9	0.3
Ripple (0) * attention (1) * Focus (1)	0.6	0.4	0.6
Ripple (0) * attention (1) * size (1)	0.6	0.4	0.6
Ripple (0) * attention (1) * size (2)	-0.1	-0.1	0.9
Ripple (0) * Focus (1) * size (1)	-0.8	-0.6	0.5
Ripple (0) * Focus (1) * size (2)	-0.3	-0.21	0.7
Attention (1) * Focus (1) * size (1)	0.73	0.5	0.5
Attention (1) * Focus (1) * size (2)	0.8	0.6	0.5
Ripple (0) * Attention (1) * Focus (1) * size (1)	-0.9	-0.7	0.4
Ripple (0) * Attention (1) * Focus (1) * size (2)	-0.7	-0.5	0.5

Using a linear regression model to predict V4 firing rate from occurrence of ripple, attention to RF vs away, size of focus of attention (narrow/wide), and size of stimulus (small/large). The digits in front the predictors represent the categories assigned to fixed effects into the model. Ripple (0 is without and 1 with ripple), attention (1 attention to RF 2 and away), focus (1 narrow and 2 wide focus of attention) and size (1 large and 2 small stimuli). The column of β reports the coefficients of each predictor estimated by model and column T is corresponding t-statistic. Asterisks indicate significance.

Table S7. Mean and SEM of firing rate across trials with and without ripple during different attention conditions in V4.

Ripple	Attention	Mean ± SEM of firing rate
No	RF	15.5 ± 0.1
No	away	13.1 ± 0.2
Yes	RF	24 ± 0.2
Yes	Away	23.1 ± 0.3

Column 1 indicates ripple occurrence in trials (with: yes; without: no ripple). Column 2 attention indicates attend to RF/away from RF condition.

Table S8. Predicting the effect of task parameters on monkey's reaction times using a linear mixed effect model.

Predictors	β	T	p-values
Ripple (0)	1.7	2.8	0.005 **
Ripple (1)	1.6	2.0	0.03 **
Ripple (2)	-1.8	-2.2	0.02**
Attention (1)	-1.6	-3.3	< 0.001***
Focus (1)	0.8	1.6	0.09
Size (1)	-1.6	-3.2	0.001**
Ripple (0) * attention (1)	-0.7	-1.3	0.19
Ripple (1) * attention (1)	0.09	0.11	0.9
Ripple (2) * attention (1)	0.1	0.1	0.8
Ripple (0) * Focus (1)	1.6	2.6	0.008***
Ripple (1) * focus (1)	-0.2	-0.3	0.75
Ripple (2) * focus (1)	0.3	0.4	0.64
Attention (1) * focus (1)	0.5	1.	0.29
Ripple (0) * size (1)	-0.2	-0.4	0.62
Ripple (1) * size (1)	-1.2	-1.6	0.10
Ripple (2) * size (1)	-1.4	-1.7	0.08
Attention (1) * size (1)	-0.9	-1.8	0.06
Focus (1) * size (1)	-5.2	-10.7	< 0.001***
Ripple (0) * attention (1) * focus (1)	0.4	0.8	0.4
Ripple (1) * attention (1) * Focus (1)	1	1.2	0.2
Ripple (2) * attention (1) * focus (1)	0.2	0.36	0.7
Ripple (0) * attention (1) * size (1)	-0.7	-1.2	0.2
Ripple (1) * attention (1) * size (1)	-1.620	-2.0	0.03
Ripple (2) * attention (1) * size (1)	0.4	0.49	0.6
Ripple (0) * focus (1) * size (1)	0.9	1.59	0.11
Ripple (1) * focus (1) * size (1)	-0.1	-0.21	0.8
Ripple (2) * focus (1) * size (1)	0.7	0.9	0.33
Attention (1) * focus (1) * size (1)	-0.2	-0.5	0.5
Ripple (0) * attention (1) * focus (1) * size (1)	1.3	2.1	0.03**
Ripple (1) * attention (1) * focus (1) * size (1)	0.7	0.88	0.3
Ripple (2) * attention (1) * focus (1) * size (1)	0.6	0.84	0.3

Results of the linear mixed effect model predicting reaction times from occurrence of ripple, attention to RF, stimulus size and narrow and wide attention. Numbers in the predictor's column indicate a category assigned to each variable. Ripple (0: no ripple, 1: ripple occurrence in V1, 2: ripple occurrence in V4, 3: ripple occurrence in V1&V4), attention (1: attention to RF, 2: attention away), focus (1: narrow and 2: wide focus of attention) and size (1: large and 2: small stimuli). Asterisks denote significance. B and T are the coefficient estimate and t-statistics.

Table S9. Mean and SEM of reaction time with respect to ripple occurrence.

Ripple	Mean ± SEM of RTs
No	310.9 ± 0.5
V1	310.9 ± 0. 8
V4	307.5 ± 0. 8
V1 & V4	305.5 ± 1. 1

Mean and SEM of reaction times are grouped by trials without ripple (no), ripple occurred in V1, V4 or both areas.

SI References

1. P. R. Roelfsema, M. Tolboom, P. S. Khayat, Different Processing Phases for Features, Figures, and Selective Attention in the Primary Visual Cortex. *Neuron* **56**, 785–792 (2007).

3. K. H. Pettersen, A. Devor, I. Ulbert, A. M. Dale, G. T. Einevoll, Current-source density estimation based on inversion of electrostatic forward solution: Effects of finite extent of neuronal activity and conductivity discontinuities. *J. Neurosci. Methods* **154**, 116–133 (2006).

4. N. K. Logothetis, C. Kayser, A. Oeltermann, In Vivo Measurement of Cortical Impedance Spectrum in Monkeys: Implications for Signal Propagation. *Neuron* **55**, 809–823 (2007).

5. V. B. Mountcastle, Modality and topographic properties of single neurons of cat's somatic sensory cortex. *J. Neurophysiol.* **20**, 408–434 (1957).

6. M. A. Kraut, J. C. Arezzo, H. G. Vaughan, Intracortical generators of the flash VEP in monkeys. *Electroencephalogr. Clin. Neurophysiol. Potentials Sect.* **62**, 300–312 (1985).

7. C. E. Schroeder, C. E. Tenke, S. J. Givre, J. C. Arezzo, H. G. Vaughan, Striate cortical contribution to the surface-recorded pattern-reversal vep in the alert monkey. *Vision Res.* **31**, 1143–1157 (1991).

8. S. J. Givre, C. E. Schroeder, J. C. Arezzo, Contribution of extrastriate area V4 to the surface-recorded flash VEP in the awake macaque. *Vision Res.* **34**, 415–428 (1994).

9. D. Ferro, J. van Kempen, M. Boyd, S. Panzeri, A. Thiele, Directed information exchange between cortical layers in macaque V1 and V4 and its modulation by selective attention. *Proc. Natl. Acad. Sci.* **118** (2021).

10. M. A. Gieselmann, A. Thiele, Stimulus dependence of directed information exchange between cortical layers in macaque V1. *eLife* **11**, e62949 (2022).

11. M. A. Gieselmann, A. Thiele, Comparison of spatial integration and surround suppression characteristics in spiking activity and the local field potential in macaque V1. *Eur. J. Neurosci.* **28**, 447–459 (2008).

12. H. Supèr, P. R. Roelfsema, Chronic multiunit recordings in behaving animals: Advantages and limitations. *Prog. Brain Res.* **147**, 263–282 (2005).
